# MVP Inhibits Influenza A Virus‐Induced Ferroptosis by Targeting IRF1 and Increasing FSP1 Activity

**DOI:** 10.1002/advs.202520371

**Published:** 2026-03-04

**Authors:** Yingbo Chen, Paili Lin, Yongfang Xia, Zhiqiang Liu, Zilu Cheng, Qingmei Zhu, Shiqi Wan, Xiaoyu Chen, Haiyan Bao, Renbo Qiao, Gechang Zhong, Ying Zhu, Shi Liu

**Affiliations:** ^1^ State Key Laboratory of Virology Modern Virology Research Center College of Life Sciences Taikang Center for Life and Medical Sciences Frontier Science Center for Immunology and Metabolism Wuhan University Wuhan China

**Keywords:** ferroptosis, FSP1, IRF1, IAV, MVP

## Abstract

Our previous studies have shown that major vault protein (MVP) is a virus‐induced host factor that participates in the innate immune response. However, little is known about the role of MVP in Influenza A virus (IAV)‐ induced ferroptosis. In this study, the expression of MVP was found to positively correlate with that of interferon regulatory factor 1 (IRF1) and ferroptosis suppressor protein 1 (FSP1), but not with glutathione peroxidase 4 (GPX4), in peripheral blood mononuclear cells from patients with IAV. In vitro and in vivo evidence indicate that MVP is a potent factor in ferroptosis resistance during IAV infection. Upon investigating the mechanisms underlying this event, MVP was found to sequester IRF1 from tumor necrosis factor receptor‐associated factor 6 (TRAF6), thereby suppressing its polyubiquitination and nuclear localization. Therefore, the transcription inhibition of IRF1 on the FSP1 promoter was removed, thereby enhancing FSP1 expression. A second wave of MVP regulation for IAV‐induced ferroptosis also occurs. In the presence of the MVP, transcriptionally induced FSP1 is released from IRF1, leading to its ubiquitination and myristoylation, which enable its recruitment to the plasma membrane, where it functions as an oxidoreductase. These findings define a ferroptosis suppression pathway during IAV infection.

## Graphical abstract

During IAV infection, MVP inhibits IRF1 polyubiquitination, thereby relieving IRF1‐mediated transcriptional inhibition of FSP1. Consequently, this leads to an upregulation of FSP1 expression, thereby reinforcing the inhibition of ferroptosis. In addition, the MVP can promote myristoylation and ubiquitination of FSP1, enabling its membrane localization and inhibiting ferroptosis.

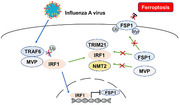

## 1 Introduction

IAV infection causes acute respiratory illness and seasonal epidemics, posing serious challenges to human and animal health [1]. IAV is a highly contagious single‐stranded RNA virus belonging to the *Orthomyxoviridae* family. After IAV infects the respiratory tract, it triggers an intense inflammatory response and extensive tissue damage by stimulating both cellular and humoral immune responses, a pathological process closely related to programmed cell death [2, 3]. Programmed cell death plays a “double‐edged sword” role in the pathogenicity of IAV. Moderate programmed cell death protects the host from IAV infection, whereas excessive cell death promotes IAV pathogenicity.

Ferroptosis is a novel form of iron‐dependent lipid peroxidation‐induced programmed cell death that differs from other forms of cell death, such as apoptosis, pyroptosis, and necrosis [4]. Ferroptosis is characterized by the excessive production of iron‐ and lipid‐reactive oxygen species (ROS) during the Fenton reaction. If not removed in time, these toxic lipid peroxides accumulate to lethal levels, damage the integrity of the cell membrane, and ultimately trigger ferroptosis [5]. However, hosts have evolved various mechanisms to monitor ferroptosis and maintain cell viability. The SLC7A11‐GSH‐GPX4 pathway is the primary signaling pathway associated with ferroptosis surveillance [6, 7]. In this signaling pathway, SLC7A11 mediates the uptake of extracellular cystine for GSH biosynthesis. Subsequently, GPX4 detoxifies lipid hydroperoxides into lipid alcohols at the expense of GSH, thereby reducing lipid peroxidation. Ubiquinone (CoQ) and ubiquinol (CoQH_2_) are radical‐trapping antioxidants that neutralize lipid peroxyl radicals. Except for the SLC7A11‐GSH‐GPX4 pathway, recent studies have shown that FSP1 and dihydroorotate dehydrogenase (DHODH) use CoQ and CoQH_2_ to suppress ferroptosis on the plasma membrane and mitochondrial membrane, respectively [8, 9, 10]. Accumulating evidence suggests that IAV infection can induce multiple forms of cell death, and the molecular mechanisms underlying these pathways vary [11, 12, 13, 14]. However, the role and mechanism of IAV‐induced ferroptosis remain unclear.

Major vault protein (MVP), also called “lung resistance‐related protein,” is the dominant structural protein of the vault complex, which is a 13‐MDa hollow barrel‐shaped ribonucleoprotein (RNP) complex found in most eukaryotes [15]. Additional evidence also confirms that MVP is closely related to other multicellular processes, such as nucleoplasmic transport, cell senescence, and apoptosis [16, 17, 18]. Our previous studies have shown that MVP is a virus‐induced host factor that participates in the innate immunity response [19]. In addition, the MVP gene is upregulated during IAV infection and inhibits IAV replication. However, a clear role for MVP in IAV‐induced cell ferroptosis has not been established. In this study, we demonstrated that MVP suppresses IAV‐induced cell ferroptosis by recruiting TRAF6, thereby inhibiting K48‐linked ubiquitination and the subsequent degradation of IRF1. Therefore, IRF1 suppresses FSP1 transcription, post‐translational modification, and plasma membrane localization, ultimately leading to ferroptosis. Our findings indicate a novel negative pathway for ferroptosis during IAV infection.

## 2 Results

### 2.1 MVP Expression Correlates with the Expression of IRF1 and FSP1 in Patients Infected with IAV

Previous studies have reported a connection between ferroptosis and IAV infection [14, 20], raising the question of how hosts use ferroptosis to regulate IAV infections. To investigate the mechanism of IAV‐induced ferroptosis, we first benchmarked ferroptosis marker levels in response to IAV infection. Using cell death and viability assays, we found that IAV A/WSN/33 (H1N1) virus infection increased cell death but decreased cell viability in a dose‐dependent manner (Figure S1A–C). To compare the forms of cell death and viability during IAV infection, a series of pharmacological approaches was employed, including ferroptosis inhibitors (Ferrostatin‐1, Fer‐1), apoptosis inhibitors (Z‐VAD‐FMK), and necroptosis inhibitors (Necrostatin‐1). Fer‐1, Z‐VAD‐FMK, or Necrostatin‐1 treatment rescued IAV‐regulated cell death and viability, suggesting that ferroptosis is as important as apoptosis and necroptosis during IAV infection (Figure S1D–F). Ferroptosis is a unique type of programmed cell death characterized by lipid peroxidation; ROS release; GSH exhaustion; and the expression of SLC7A11, FSP1, prostaglandin‐endoperoxide synthase 2 (PTGS2), acyl‐CoA synthetase long‐chain family member 4 (ACSL4), and glutathione‐specific gamma‐glutamylcyclotransferase 1 (CHAC1) [4]. As expected, intracellular lipid peroxidation and Fe^2+^ levels were enhanced, whereas GSH levels were reduced in response to IAV infection (Figure S1G,H). Ferrostatin‐1 treatment inhibited IAV‐mediated induction of ferroptosis features (Figure S1G,H). In this vein, CHAC1 and ACSL4 expression increased, whereas FSP1 and SLC7A11 expression decreased during IAV infection (Figure S1I–L). Intriguingly, IAV infection did not affect GPX4 expression, suggesting that GPX4‐mediated ferroptosis was not the main pathway during IAV infection (Figure S1M). Next, we investigated whether IAV induces ferroptosis in vivo. IAV infection increased Fe^2^^+^ concentration, 4‐hydroxynonenal (4‐HNE) staining, and *Ptgs2* expression, but ferrostatin‐1 treatment inhibited IAV‐induced Fe^2^^+^ concentration, 4‐HNE staining, and *Ptgs2* expression (Figure S1N–Q). In this manuscript, to avoid confusion between murine and human genes, the names of murine genes are italicized, with the first letter capitalized and the names of murine and human proteins, as well as human genes, have been capitalized.

**FIGURE 1:**
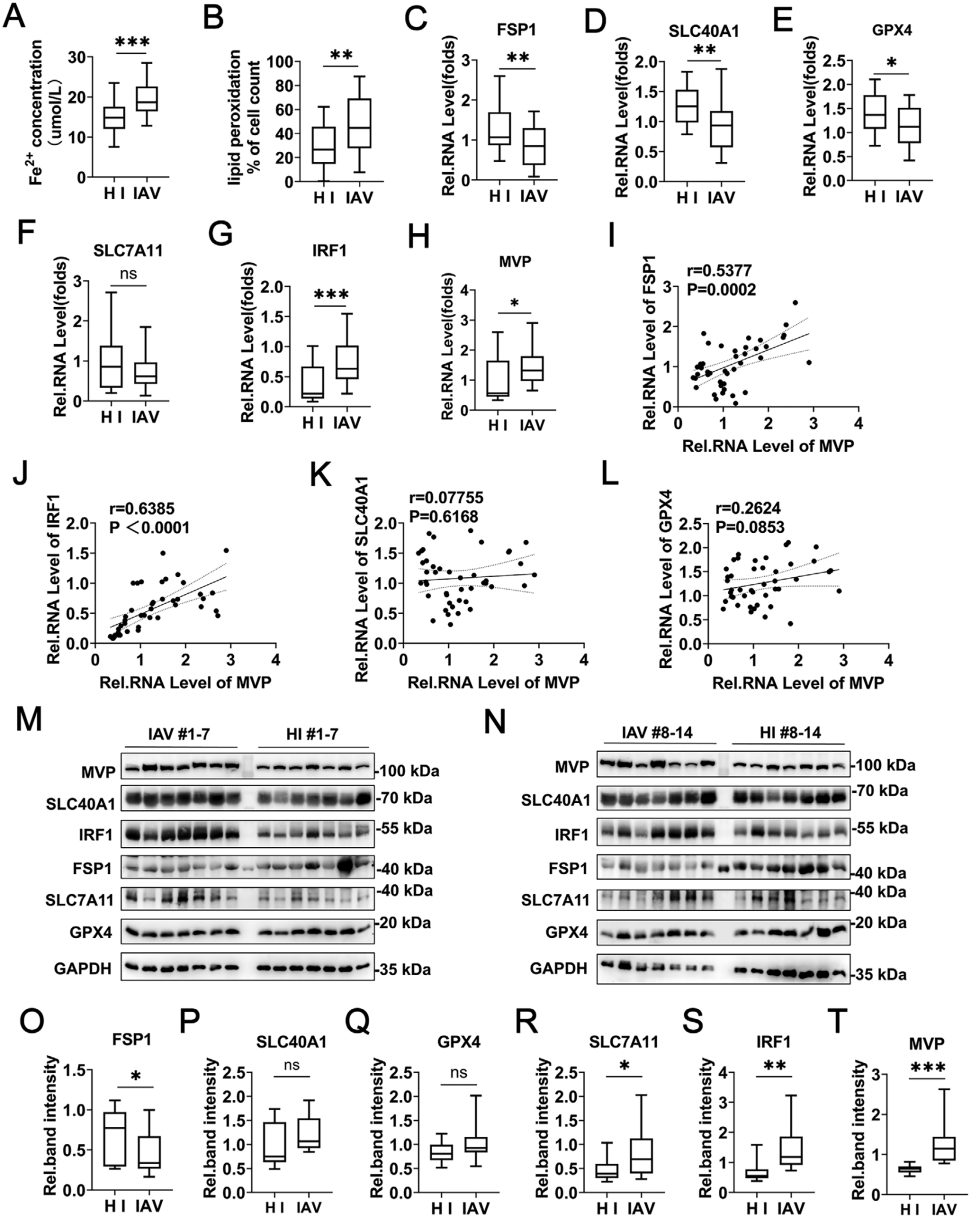
Analysis of ferroptosis, MVP, and IRF1 expression in patients infected with IAV. (A) Serum Fe^2+^ concentration in patients infected with IAV (*n* = 24) and healthy individuals (HI) (*n* = 20). (B–H) PBMCs were collected from patients infected with IAV (*n* = 24) and HI (*n* = 20). Lipid peroxidation levels were measured using BODIPY 581/591 C11 staining and flow cytometry (B). The mRNA levels of FSP1 (C), SLC40A1 (D), GPX4 (E), SLC7A11(F), IRF1 (G), and MVP (H) were measured using qPCR. Data are expressed as mean ± SEM. Boxplots show medians with 25% and 75% as well as error bars for 5% and 95% percentiles. (I–L) The mRNA levels of MVP and mRNA levels of FSP1(I), SLC40A1(J), GPX4(K), or IRF1(L) in patients infected with IAV as analyzed by Pearson's correlation analysis. (M,N) PBMCs were collected from patients infected with IAV (*n* = 14) and HI (*n* = 14). Protein levels of the indicated ferroptosis‐related genes were measured by Western blot. (O–T) The relative protein abundance in (M and N) was quantified by densitometric analysis of the blots and normalized to their respective GAPDH. Data are expressed as mean ± SEM. Boxplots show medians with 25% and 75% as well as error bars for 5% and 95% percentiles. We acknowledge the use of GraphPad Prism 8.0 (GraphPad Software, San Diego, CA) and Adobe Photoshop CC2019 (Adobe Inc., San Jose, CA) for generating this figure. Statistical significance was assessed using the two‐tailed Student's *t*‐test. *p < 0.05; **p < 0.01; ***p < 0.001; n.s. = not significant. See also Figure S1.

To elucidate the mechanism of IAV‐regulated ferroptosis, we obtained blood samples from healthy individuals (HI) and from patients diagnosed with IAV infection. Two independent HI and patients infected with IAV cohorts were studied. Cohort #1 comprised peripheral blood mononuclear cells (PBMCs) and serum from HI (*n* = 20) and patients infected with IAV (*n* = 24). Cohort #2 consisted of PBMCs from HI (*n* = 14) and patients infected with IAV (*n* = 14). Consistent with the above in vitro and in vivo data, the Fe^2+^ concentration in the serum and lipid peroxidation levels in PBMCs were higher in patients with IAV than in HI in Cohort #1 (Figure 1A,B). Next, we investigated the expression of ferroptosis markers in the PBMCs from Cohort #1 using a qPCR assay. Accordingly, the mRNA levels of FSP‐1 and SLC40A1 were impaired in patients infected with IAV compared with HI (Figure 1C, D). However, the mRNA levels of GPX4 and SLC7A11 were mildly altered in patients infected with IAV compared to HI, again validating that GPX4 does not participate in IAV‐regulated ferroptosis (Figure 1E,F). We and others have previously reported that the MVP and IRF1 are two important genes in innate immunity and inflammation during IAV infection [21, 22]. Consistently, the mRNA levels of MVP and IRF1 were higher in patients infected with IAV than in HI (Figure 1G,H). Elevated MVP expression in IAV‐infected patients positively correlated with the expression of FSP1 and IRF1, but not with the expression of GPX4 and SLC40A1 (Figure 1I–L). In Cohort #2, above, ferroptosis marker protein levels were assessed using Western blot. Results showed that FSP1 protein levels were reduced, while MVP and IRF1 protein levels were induced in patients infected with IAV compared with HI (Figure 1M–T). As expected, protein levels of GPX4 and SLC7A11 were mildly altered in patients infected with IAV compared to HI (Figure 1M–T). Collectively, these findings demonstrate that the MVP/IRF1/FSP1 axis plays an important role in ferroptosis during IAV infection.

### 2.2 MVP is a Potent Ferroptosis Suppressor of IAV‐Induced Ferroptosis

**FIGURE 2:**
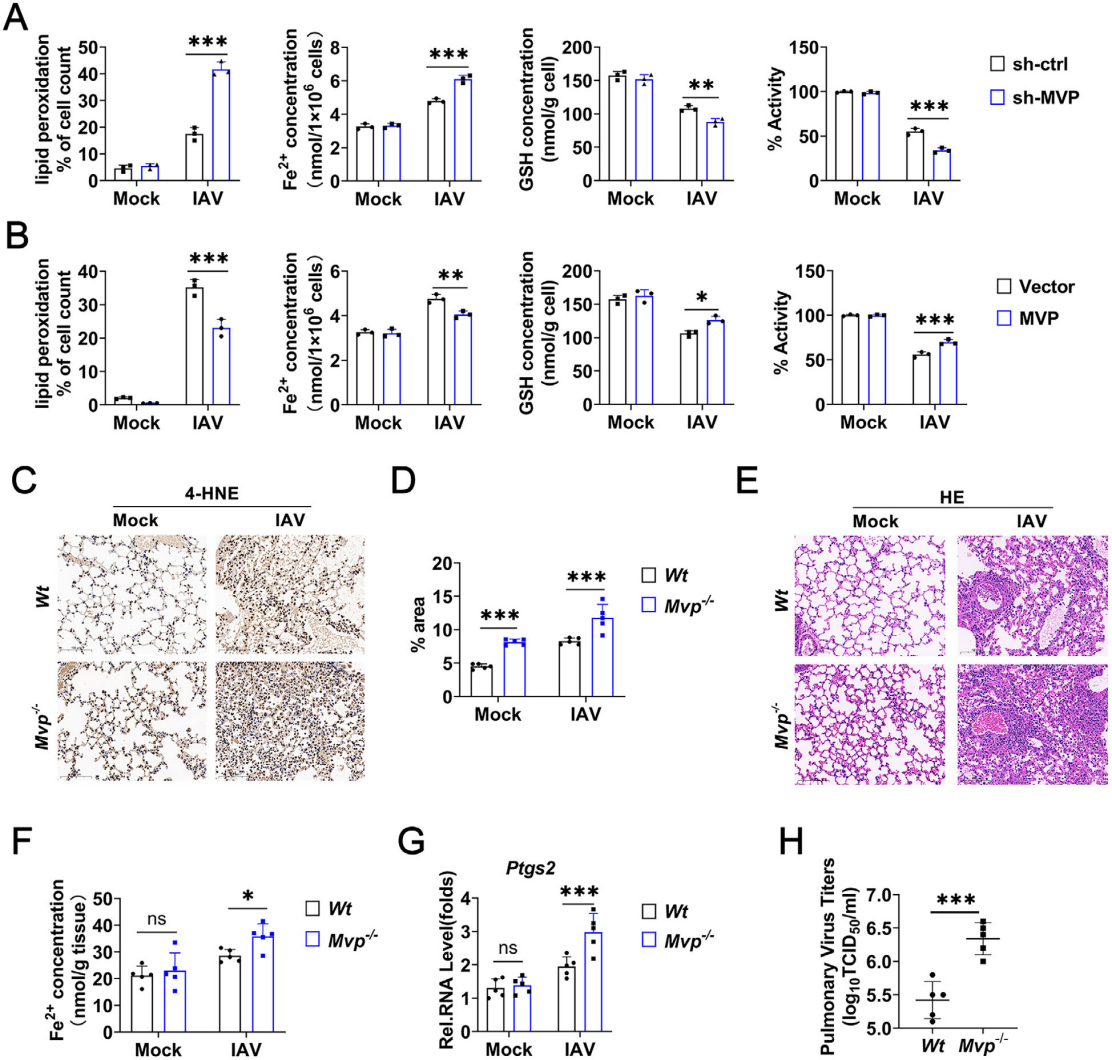
MVP inhibits IAV‐induced ferroptosis in vitro and in vivo. (A) A549 cells were transfected with sh‐control or sh‐MVP for 36 h and infected with or without IAV (MOI = 0.1) for 12 h, followed by measuring lipid peroxidation levels, Fe^2+^ concentrations, GSH levels, and cell viability. (B) Experiments were similar to those in (A), except cells were transfected with vector control or pCMV‐MVP. (C–E) Wild‐type (*Wt*) and *Mvp^−/−^* mice were infected with IAV (1 × 10^4^ PFU) and/or intraperitoneally injected with ferrostatin‐1 (10 mg/kg, once every 2 days) for 4 days. Representative histopathological changes in 4‐HNE‐stained and H&E‐stained lung tissues. Graphs depict quantification of the 4‐HNE‐stained area. Scale bars: 100 µm, *n* = 5 randomly selected magnification fields. (F–H) Experiments were similar to those in (C), except that Fe^2+^ concentration (F), Ptgs2 mRNA levels (G), and viral titers (H) in the lung were measured. Scale bars: 100 µm. We acknowledge the use of GraphPad Prism 8.0, Adobe Photoshop CC2019, and SlideViewer 2.5 (3DHISTECH Ltd., Hungary) for generating this figure. All experiments were performed in triplicate. The data are presented as mean ± SD. Statistical significance was assessed using two‐way ANOVA analysis in (A, B, D, F, and G), two‐tailed Student's *t*‐test in (H) for comparisons. *p < 0.05; **p < 0.01; ***p < 0.001; n.s. = not significant. See also Figure S2.

To investigate the effect of MVP on IAV‐induced ferroptosis in vitro, we constructed a pCMV‐MVP overexpression plasmid and MVP‐specific short hairpin RNA (shRNA) and verified their efficiency (Figure S2A–D). shRNA‐MVP#3 was selected for MVP knockdown as described below. Consistent with our previous results [23], IAV infection induced the mRNA and protein levels of MVP in a dose‐dependent manner (Figure S2E–G). By contrast, MVP knockdown increased IAV replication (Figure S2H–K). Next, we examined the effect of MVP on IAV‐induced ferroptosis. MVP knockdown increased IAV‐induced lipid peroxidation and Fe^2+^ accumulation, whereas it decreased IAV‐inhibited GSH levels and cell viability (Figure 2A and Figure S2L). In contrast, MVP overexpression inhibited IAV‐induced lipid peroxidation and Fe^2+^ accumulation, while enhancing IAV‐inhibited GSH levels and cell viability (Figure 2B and Figure S2M).

To examine the effect of MVP on IAV‐induced ferroptosis in vivo, we used the MVP‐knockout (*Mvp^−/−^*) mouse model as previously described [24]. Compared to wild‐type (*Wt*) mice, *Mvp^−/−^* mice exhibited higher levels of Fe^2+^, 4‐HNE staining, and pulmonary inflammation in the lung tissues during IAV infection (Figure 2C–F). As expected, levels of *Ptgs2* mRNA and IAV viral titers were higher in the lung tissues of *Mvp^−/−^* mice than in *Wt* mice during IAV infection (Figure 2G,H). In summary, these findings demonstrate that MVP is a negative regulator of IAV‐induced ferroptosis.

### 2.3 MVP Negatively Regulates Ferroptosis by Interacting with IRF1

**FIGURE 3:**
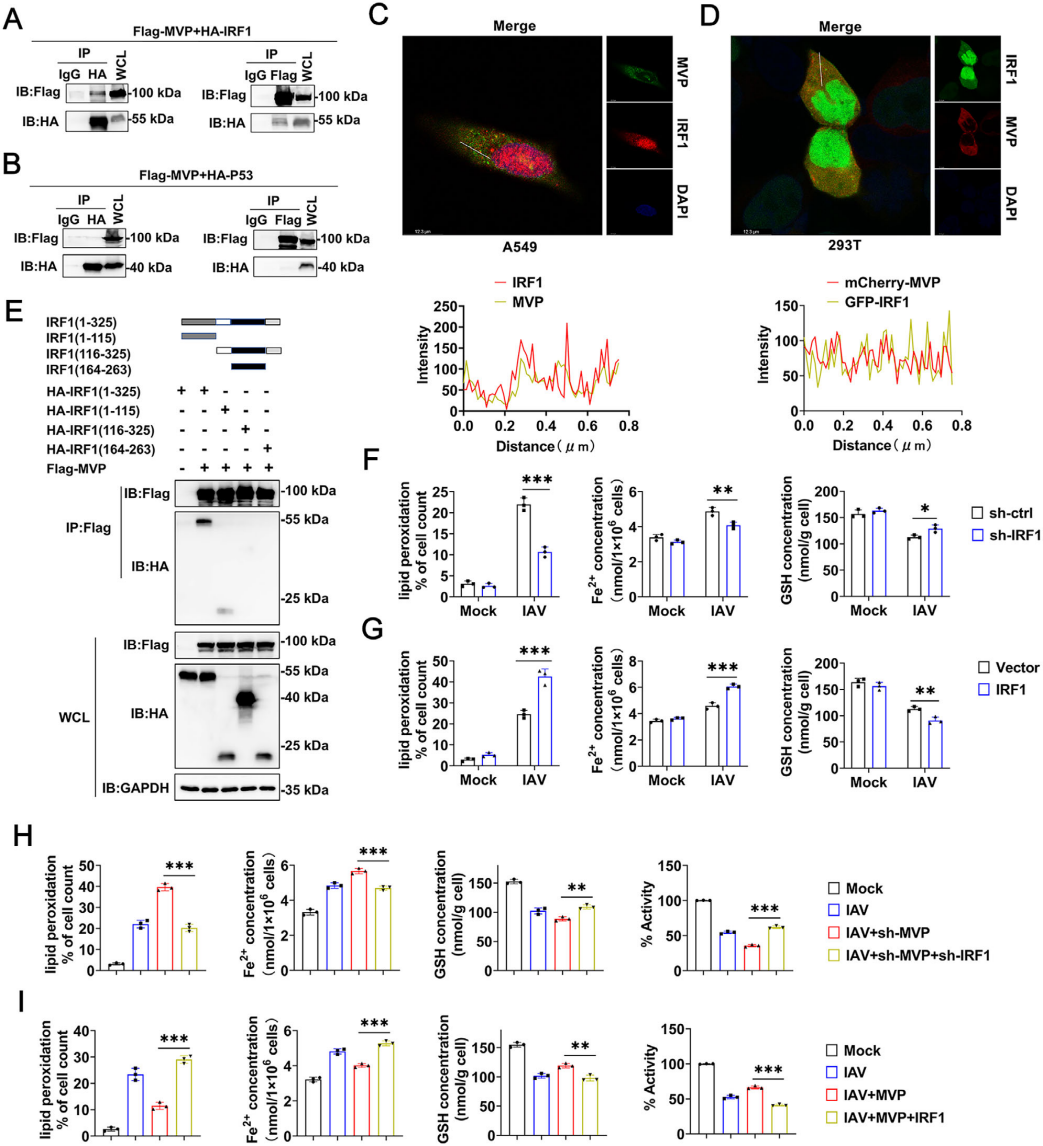
MVP associates with IRF1 to inhibit IAV‐induced ferroptosis. (A) 293T cells were transfected with Flag‐MVP and HA‐IRF1 for 48 h. Co‐immunoprecipitation (Co‐IP) and immunoblot analyses were performed with the indicated antibodies. (B) Experiments were similar to those in (A), except that cells were transfected with HA‐p53. (C) A549 cells were infected with IAV (MOI = 0.1) for 12 h and subjected to an immunofluorescence assay (IFA). Line intensity plots show colocalization between MVP and IRF1. Scale bars, 12.3 µm. (D) 293T cells transfected with mCherry‐MVP and GFP‐IRF1 plasmids for 36 h and subjected to IFA. Line intensity plots show colocalization between MVP and IRF1. Scale bars, 12.3 µm. (E) Domain mapping of the IRF1 and MVP interaction. 293T cells were transfected with the indicated plasmid for 48 h. Co‐IP and immunoblot analyses were performed with the indicated antibodies. At the top is a schematic diagram of IRF1 truncation. (F) A549 cells were transfected with sh‐ctrl or sh‐IRF1 for 36 h and infected with or without IAV (MOI = 0.1) for 12 h, followed by measuring lipid peroxidation levels, Fe^2+^ concentrations, and GSH levels. (G) Experiments were similar to those in (F), except cells were transfected with vector control or pCMV‐IRF1. (H) A549 cells were transfected with the indicated shRNAs for 36 h and infected with or without IAV (MOI = 0.1) for 12 h, followed by measuring lipid peroxidation levels, Fe^2+^ concentrations, GSH levels, and cell viability. (I) Experiments were similar to those in (H), except cells were transfected with the indicated plasmids. We acknowledge the use of GraphPad Prism 8.0, Adobe Photoshop CC2019, and LAS X (version 3.7.4.23463; Leica Inc.) for generating this figure. All experiments were performed in triplicate. The data are presented as mean ± SD. Statistical significance was assessed using two‐way ANOVA analysis in (F and G), two‐tailed Student's *t*‐test in H and I for comparisons. *p < 0.05; **p < 0.01; ***p < 0.001. n.s. = not significant. See also Figure S3.

Our previous study demonstrated that MVP promoted hepatocellular carcinoma by associating with IRF2 [18]. Within the IRF family, IRF1 has been widely reported to regulate ferroptosis [25, 26, 27]. In this study, we found that MVP expression correlates with IRF1 expression in the PBMCs of patients with IAV. Thus, we speculated that MVP interacts with IRF1 in response to IAV infection. Co‐immunoprecipitation (Co‐IP) and reverse Co‐IP experiments revealed that Flag‐tagged MVP interacted with HA‐tagged IRF1 (Figure 3A). In our previous study, we found that MVP did not interact with p53 [18]. Thus, p53 was included as a negative control for comparison (Figure 3B). Using immunofluorescence staining, we also showed that MVP co‐localized with IRF1 in A549 and 293T cells (Figure 3C,D). To map the region of IRF1 that interacts with MVP, we constructed a series of IRF1truncation mutants. We demonstrated that the DNA‐binding domain (DBD) of IRF1 (amino acids 1‐115) is required for its interaction with MVP (Figure 3E).

Because MVP is associated with IRF1, we examined the role of the MVP/IRF1 axis in IAV‐induced ferroptosis. IAV infection increased the mRNA and protein expression levels of IRF1 in a dose‐dependent manner (Figure S3A,B). To investigate the effect of IRF1 on IAV‐induced ferroptosis, we constructed a pCMV‐IRF1 overexpression plasmid and IRF1‐specific shRNAs and tested their efficiency (Figure S3C–F). shRNA‐IRF1#1 was selected for the knockdown of IRF1 described below. The results indicated that IRF1 knockdown decreased Fe^2+^ levels and lipid peroxidation, but increased GSH levels and cell viability during IAV infection (Figure 3F and Figure S3G,H). Conversely, IRF1 overexpression elevated Fe^2+^ and lipid peroxidation levels, but suppressed GSH levels and cell viability during IAV infection (Figure 3G and Figure S3I,J). Interestingly, IRF1 knockdown abolished the effect of MVP shRNA on lipid peroxidation levels, Fe^2+^ levels, GSH levels, and cell viability during IAV infection (Figure 3H and Figure S3K). By contrast, IRF1 overexpression recovers the inhibitory effect of MVP on lipid peroxidation levels and Fe^2+^ levels during IAV infection, indicating that IRF1 is a downstream signaling molecule in MVP‐mediated signaling (Figure 3I and Figure S3L). Collectively, these findings demonstrate that IAV infection promotes the association of MVP with IRF1, thereby inhibiting IRF1's pro‐ferroptosis function.

### 2.4 MVP Inhibits Polyubiquitination of IRF1 via Disrupting IRF1/TRAF6 Interactions

**FIGURE 4:**
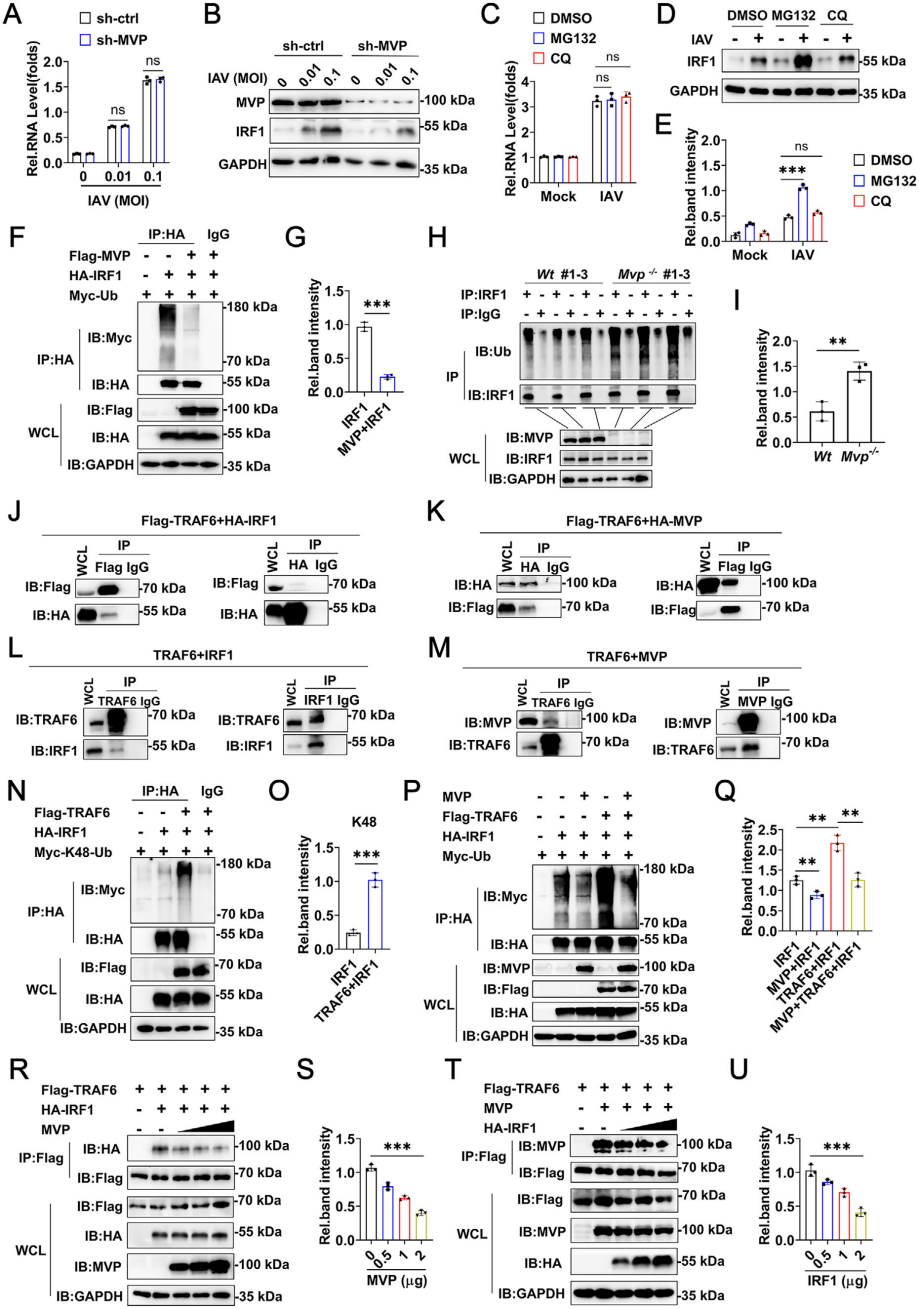
MVP inhibits K48‐linked polyubiquitination of IRF1 via competitive binding to TRAF6. (A,B) A549 cells were infected with or without the indicated dose of IAV and/or transfected with sh‐control or sh‐MVP for 12 h, and subjected to qPCR (A) and Western blot (B) assays. (C–E) A549 cells were infected with or without IAV (MOI = 0.1) for 12 h, then treated with MG132 (10 µm) or CQ (10 µm) for 4 h, followed by qPCR (C) and Western blot (D) assays. The relative intensity of IRF1 was measured using ImageJ and normalized to its respective GAPDH (E). (F,G) 293T cells were transfected with the indicated plasmids for 36 h and treated with MG132 (10 µm) for 4 h. Co‐IP and immunoblot analyses were performed with the indicated antibodies (F). The relative intensity of polyubiquitination of IRF1 was measured using ImageJ and normalized to the respective unmodified IRF1 (G). (H,I) Wild‐type (*Wt*) and *Mvp^−/−^* mice were infected with IAV (1 × 10^4^ PFU) for 4 days. Lung tissue was collected and subjected to Co‐IP and Western blot analysis (H). The relative intensity of polyubiquitination of IRF1 was measured using ImageJ and normalized to the respective unmodified IRF1 (I). (J,K) 293T cells were transfected with the indicated plasmids for 48 h. Co‐IP and immunoblot analyses were performed with the indicated antibodies. (L,M) Experiments were performed similarly to those in (H), except that the indicated antibodies were used. (N–Q) Experiments were similar to those in (F), except that Flag‐TRAF6 was used. (R, S) 293T cells were transfected with Flag‐TRAF6 (2 µg), HA‐IRF1 (2 µg), or increasing amounts of MVP (0.5, 1, 2 µg) expression plasmids for 48 h and treated with MG132 (10 µm) for 4 h. Co‐IP and immunoblot analyses were performed with the indicated antibodies (R). The relative intensity of HA‐IRF1 in IP was measured using ImageJ and normalized to its respective HA‐IRF1 in WCL (S). (T,U) 293T cells were transfected with Flag‐TRAF6 (2 µg), MVP (2 µg), or increasing amounts of HA‐IRF1 (0.5, 1, 2 µg) expression plasmids for 48 h and treated with MG132 (10 µm) for 4 h. Co‐IP and immunoblot analyses were performed with the indicated antibodies (T). The relative intensity of MVP in IP was measured using ImageJ and normalized to its respective MVP in WCL (U). We acknowledge the use of GraphPad Prism 8.0 and Adobe Photoshop CC2019 for generating this figure. All experiments were performed in triplicate. The data are presented as mean ± SD. Statistical significance was assessed using two‐way ANOVA analysis in (A, C, and E), two‐tailed Student's *t*‐test in (G, I, O, Q, S, and U) for comparisons. *p < 0.05; **p < 0.01; ***p < 0.001. n.s. = not significant. See also Figure S4.

Since MVP is associated with IRF1, we next investigated the role of MVP on IRF1 expression. The MVP knockdown did not affect IRF1 RNA levels but decreased IRF1 protein levels (Figure 4A,B). We speculate that MVP regulates IRF1 stability via the ubiquitin‐proteasomal system. As expected, the proteasome inhibitor MG132 increased IAV‐induced IRF1 protein levels, but not IAV‐induced IRF1 mRNA levels (Figure 4C–E). The autophagy inhibitor Chloroquine (CQ) was included as a negative control for comparison (Figure 4C–E). In the overexpression system, MVP inhibited the polyubiquitination of IRF1 (Figure 4F,G). In this vein, IAV gave rise to lower levels of IRF1 polyubiquitination in *Mvp^−/−^* mice compared with *Wt* mice (Figure 4H,I). Using UbiBrowser, we identified TRAF6 as a potential E3 ubiquitin ligase responsible for IRF1 polyubiquitination (Figure S4A). Co‐IP and reverse Co‐IP experiments revealed that TRAF6 interacted with both MVP and IRF1 (Figure 4J,K). Similar results were obtained using the IAV‐infected mouse model (Figure 4L,M). Further experiments have shown that TRAF6 enhanced K48‐linked, but not K63‐linked or K48R‐linked polyubiquitination of IRF1 (Figure 4N,O, and Figure S4B–E). Interestingly, MVP inhibited TRAF6‐mediated IRF1 polyubiquitination (Figure 4P,Q). To explore the mechanisms underlying this event, competitive co‐IP experiments were performed. As shown in Figure 4R,S, transfection with increments of plasmid encoding MVP disrupted TRAF6 and IRF1 interactions. In contrast, IRF1 disrupted MVP/TRAF6 interactions in a dose‐dependent manner (Figure 4T,U). In summary, these findings demonstrate that MVP inhibits IRF1 polyubiquitination by competitively binding TRAF6.

### 2.5 The MVP/IRF1 Axis Regulates FSP1 Expression and Localization During IAV Infection

Since MVP associates with IRF1 to regulate IAV‐induced ferroptosis, we investigated which ferroptosis molecule is involved in the signaling downstream of the MVP/IRF1 axis. The ENCODE chromatin immunoprecipitation (ChIP)‐seq database showed that the FSP1 promoter has two potential IRF1 binding sites (RE1‐2) (Figure S5A). Consistently, IRF1 knockdown increased the mRNA and protein levels of FSP1. Simultaneously, the overexpression of IRF1 decreased the mRNA and protein levels of FSP1 during IAV infection (Figure S5B–E). To investigate the role of MVP and IRF1 in FSP1 regulation, 293T cells were co‐transfected with MVP/IRF1‐overexpressing plasmids and pFSP1‐Luc, which includes the FSP1 promoter region spanning from −2000 to +100 bp. Luciferase activity assays indicated that IRF1 overexpression reduces FSP1 promoter activity, whereas MVP overexpression increases it (Figure S5F). The ChIP‐qPCR assay showed that IRF1 bound to RE1 and RE2 of the FSP1 promoter, whereas MVP inhibited IRF1 binding to the FSP1 promoter (Figure S5G–I). These data suggested that IRF1 is a negative regulator of FSP1 transcription.

**FIGURE 5:**
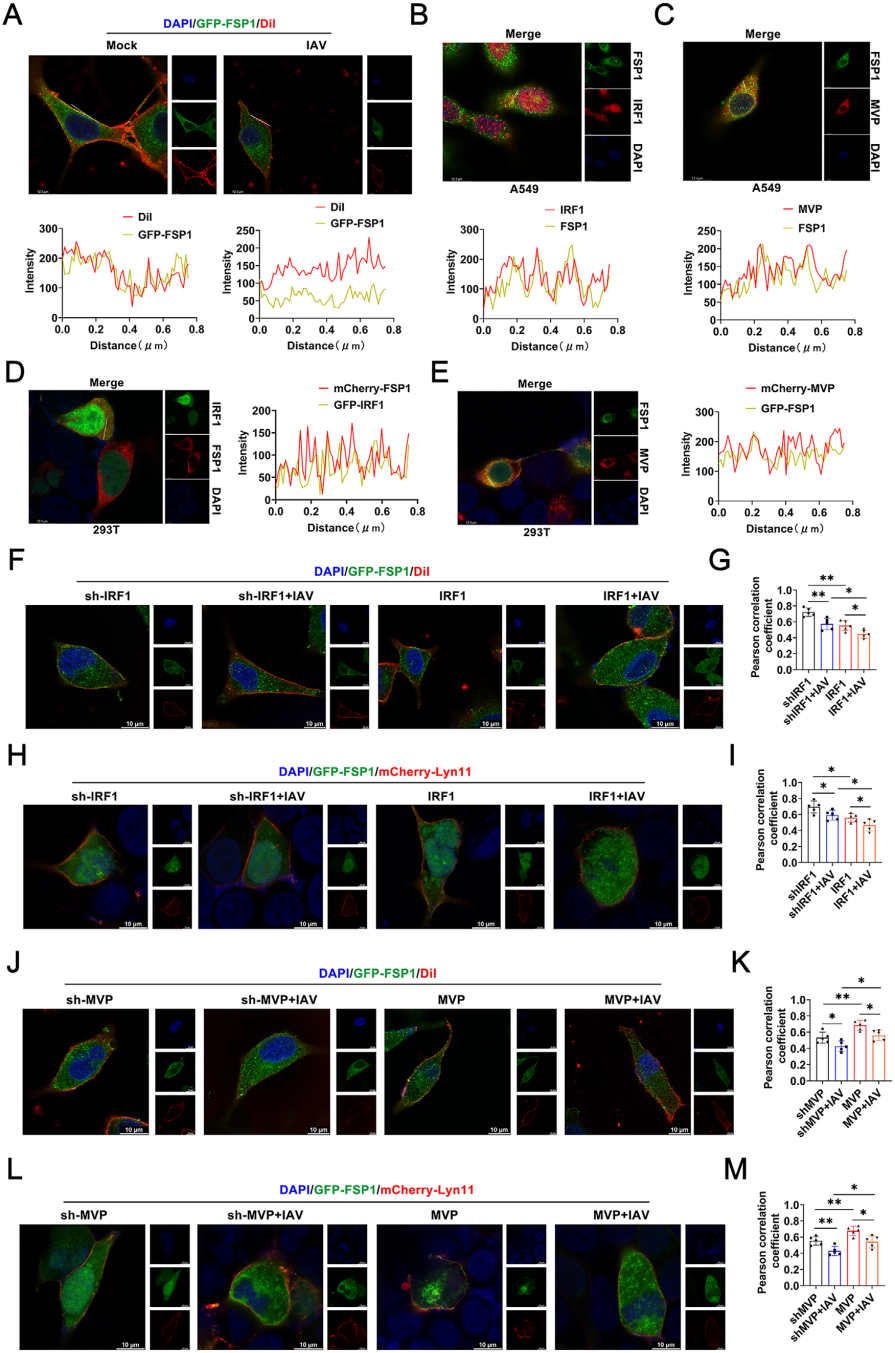
The MVP/IRF1 axis regulates FSP1 expression and localization during IAV infection. (A) A549 cells were transfected with the indicated plasmids for 36 h and infected with or without IAV (MOI = 0.1) for 12 h. Then, cells were treated with DiI (10 µm) for 20 min before the Immunological Fluorescence Assay (IFA). (B,C) A549 cells were infected with or without IAV (MOI = 0.1) for 12 h before IFA. (D,E) A549 cells were transfected with the indicated plasmids 36 h prior to IFA. (F,G) A549 cells were transfected with GFP‐FSP1, sh‐IRF1/or pCMV‐IRF1 for 36 h and infected with or without IAV (MOI = 0.1) for 12 h. Then, cells were treated with DiI (10 µm) for 20 min prior to IFA (F). Colocalization was analyzed using ImageJ (G). Scale bar: 10 µm. (H,I) 293T cells were transfected with GFP‐FSP1, mCherry‐Lyn11, sh‐IRF1/or pCMV‐IRF1 for 36 h, then infected with or without IAV (MOI = 0.1) for 12 h prior to IFA (H). Colocalization was analyzed using ImageJ (I). Scale bar: 10 µm. (J–M) Experiments were performed similar to those in (F–I), except that sh‐MVP or/and pCMV‐MVP was used. Scale bar: 10 µm. We acknowledge the use of GraphPad Prism 8.0, Adobe Photoshop CC2019, and LAS X for generating this figure. Line intensity plots show the colocalization of proteins. All experiments were performed in triplicate. The data are presented as mean ± SD. Statistical significance was assessed using two‐tailed Student's *t*‐test for comparisons. *p < 0.05; **p < 0.01; ***p < 0.001. n.s. = not significant. See also Figure S5.

Previous studies have indicated that the subcellular localization of FSP1 determines its ability to suppress ferroptosis [8, 10]. To explore whether IAV infection influences the subcellular localization of FSP1, we used the lipophilic tracer DiI, which labeled the plasma membrane. FSP1 was located at the cytomembrane in uninfected cells, but its cell membrane localization of FSP1 decreased after IAV infection (Figure 5A). Surprisingly, FSP1 co‐localized with MVP and IRF1 during IAV infection (Figure 5B,C). Similar results were obtained for transfection with GFP‐FSP1, mCherry‐IRF1, or mCherry‐MVP (Figure 5D,E). Interestingly, IRF1 knockdown promoted FSP1 translocation to the plasma membrane. IRF1 overexpression restricted FSP1 translocation to the plasma membrane (Figure 5F,G). Similar results were obtained with mCherry‐Lyn11, a protein confirmed to be located at the cytomembrane (Figure 5H,I). Meanwhile, MVP knockdown restricted FSP1 translocation to the plasma membrane. In contrast, MVP overexpression promoted FSP1 translocation to the plasma membrane (Figure 5J–M).

Given that immunofluorescence staining experiments suggested that FSP1 co‐localized with MVP and IRF1 during IAV infection, we suspected that MVP and IRF1 compete with FSP1 for binding. Consistent with the immunofluorescence staining results, Co‐IP and reverse Co‐IP experiments revealed that both IRF1 and MVP interacted with FSP1 (Figure S5J,K). Competitive Co‐IP experiments demonstrated that IRF1 disrupted MVP/FSP1 interactions, and MVP disrupted IRF1/FSP1 interactions (Figure S5L,M). Endogenous Co‐IP results suggested that MVP was associated with IRF1 in unstimulated cells, and this association was disrupted 24 h after IAV infection (Figure S5N). In contrast, MVP was weakly associated with FSP1 in unstimulated cells, and this association increased after IAV stimulation (Figure S5N). Collectively, these findings demonstrate that MVP/IRF1 regulates FSP1 function via two mechanisms. The first is that MVP promotes FSP1 expression by ubiquitinating IRF1 for degradation. Consequently, IRF1 is unable to bind to the FSP1 promoter. The second mechanism is MVP‐promoted FSP1 translocation to the plasma membrane, which is achieved by abolishing the interaction between FSP1 and IRF1.

### 2.6 The MVP/IRF1 Axis Affects the Localization of FSP1 by Regulating the Ubiquitination and Myristoylation of FSP1

**FIGURE 6:**
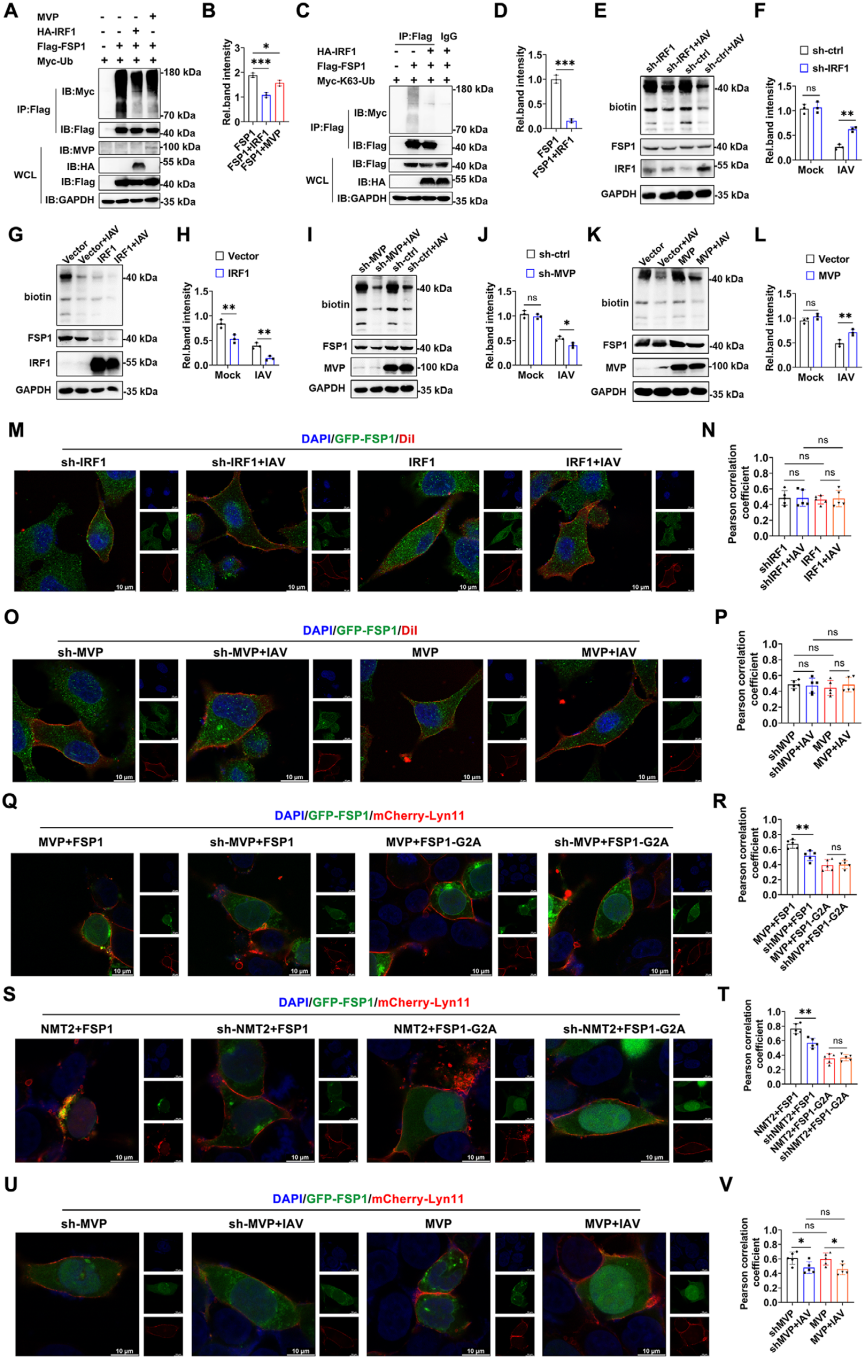
The MVP/IRF1 axis regulated the ubiquitination and myristoylation of FSP1. (A,B) 293T cells were transfected with the indicated plasmids for 36 h, then treated with MG132 (10 µm) for 4 h. Co‐IP and immunoblot analyses were performed with the indicated antibodies (A). The relative intensity of polyubiquitination of FSP1 was measured using ImageJ and normalized to their respective unmodified FSP1 (B). (C,D) Experiments were performed similarly to those in (A and B), except that Myc‐K63‐Ub was used. (E–L) A549 cells were transfected with the indicated plasmids or shRNAs for 36 h and infected with or without IAV (MOI = 0.1) for 12 h, followed by click chemistry analyses. The quantification of the Western blots is shown in the adjacent graphs. (M,N) A549 cells were transfected with the indicated plasmids or shRNAs for 36 h and treated with iNMT (10 µm) and infected with or without IAV for 12 h. Then, cells were treated with DiI (10 µm) for 20 min before IFA. Colocalization was analyzed using ImageJ (N): scale bars, 10 µm. (O,P) Experiments were performed similarly to those in (M,N), except that sh‐MVP and pCMV‐MVP were used. (Q,R) 293T cells were transfected with GFP‐FSP1/or GFP‐FSP1‐G2A, mCherry‐Lyn11, sh‐MVP/or pCMV‐MVP for 36 h, then infected with or without IAV (MOI = 0.1) for 12 h prior to IFA (Q). Colocalization was analyzed using ImageJ (R); scale bars 10 µm. (S,T) Experiments were performed similarly to those in (Q,R), except that sh‐NMT2 or/and pCMV‐NMT2 was used. (U,V) 293T cells were transfected with GFP‐FSP1, mCherry‐Lyn11, sh‐NMT2, sh‐MVP/or pCMV‐MVP for 36 h and infected with or without IAV (MOI = 0.1) for 12 h prior to IFA (U). Colocalization was analyzed using ImageJ (V): scale bars, 10 µm. We acknowledge the use of GraphPad Prism 8.0, Adobe Photoshop CC2019, and LAS X for generating this figure. All experiments were performed in triplicate. The data are presented as mean ± SD. Statistical significance was assessed using two‐way ANOVA analysis in (F, H, J, and L), two‐tailed Student's *t*‐test in (B, D, N, P, R, T, and V) for comparisons. *p < 0.05; **p < 0.01; ***p < 0.001. n.s. = not significant. See also Figure S6.

Previous studies have shown that FSP1 myristoylation and TRIM21‐mediated K63‐linked ubiquitination affect its cell membrane localization [8, 10, 28]. Next, we sought to determine whether the MVP/IRF1 axis regulates the ubiquitination and myristoylation of FSP1. IRF1 overexpression decreased the ubiquitination of FSP1, whereas MVP overexpression mildly decreases the ubiquitination of FSP1 (Figure 6A,B). Studies showed that IRF1 overexpression inhibited the K63‐linked ubiquitination of FSP1 (Figure 6C,D). The myristoylation of FSP1 was detected using click chemistry. IAV infection inhibited FSP1 myristoylation (Figure S6A,B). An N‐myristoyltransferase (NMT) inhibitor (ddd85646, iNMT) was used as a positive control (Figure S6A,B). This experiment demonstrated that IAV reduced FSP1 myristoylation, which was reversed by IRF1 knockdown (Figure 6E,F). In contrast, IRF1 overexpression inhibited FSP1 myristoylation, and IRF1 overexpression and IAV infection synergistically inhibited FSP1 myristoylation (Figure 6G,H). MVP knockdown synergistically inhibited IAV, reducing FSP1 myristoylation (Figure 6I,J). Moreover, IAV‐reduced FSP1 myristoylation was recovered by MVP overexpression (Figure 6K,L). Of note, iNMT treatment abolishes the effects of MVP, IRF1, and IAV on FSP1 membrane localization (Figure 6M–P).

We next investigated the mechanism by which the MVP/IRF1 axis regulates FSP1 myristoylation and ubiquitination. FSP1 interacted with NMT2 and TRIM21, but not NMT1 (Figure S6C–E). Similarly, IRF1 and MVP also interacted with NMT2 and TRIM21, but not NMT1 (Figure S6F–K). Previous studies have shown that the FSP1 point mutation (G2A) is unable to be myristoylated [8, 10]. MVP knockdown restrained FSP1 translocation to the plasma membrane, while MVP overexpression promoted FSP1 translocation to the plasma membrane (Figure 6Q,R). However, MVP did not affect FSP1‐G2A translocation to the plasma membrane (Figure 6Q,R). Consistently, NMT2 knockdown restrained FSP1 translocation to the plasma membrane, while NMT2 overexpression promoted FSP1 translocation to the plasma membrane (Figure 6S,T). However, NMT2 did not affect FSP1‐G2A translocation to the plasma membrane (Figure 6S,T). Further study showed that knockdown or overexpression of MVP has no significant effect on FSP1 translocation in NMT2 knockdown cells, indicating that MVP promotes FSP1 translocation to the plasma membrane via NMT2 (Figure 6U,V).

We investigated the relationship between the myristoylation and ubiquitination of FSP1. TRIM21 knockdown inhibited the myristoylation of FSP1 (Figure S6L,M). Previous studies have shown that the FSP1 point mutation (K366R) is unable to be ubiquitinated [28]. As expected, the myristoylation of FSP1 was inhibited in FSP1 K366R‐transfected cells compared to that in FSP1 WT‐transfected cells (Figure S6N,O). Next, we investigated the effect of FSP1 myristoylation on its ubiquitination. Neither iNMT treatment nor NMT2 knockdown affected the ubiquitination of FSP1 (Figure S6P–S). Consistently, FSP1 G2A cannot affect the ubiquitination of FSP1 (Figure S6T,U). Collectively, these findings demonstrate that by competitively binding to IRF1, MVP recruits TRIM21 and FSP1, leading to FSP1 ubiquitination. Then, ubiquitinated FSP1 interacts with NMT2, leading to FSP1 myristoylation. As a result, FSP1 translocates to the membrane (Figure S6V).

### 2.7 The MVP/IRF1 Axis‐Regulated IAV‐Induced Ferroptosis via FSP1

**FIGURE 7:**
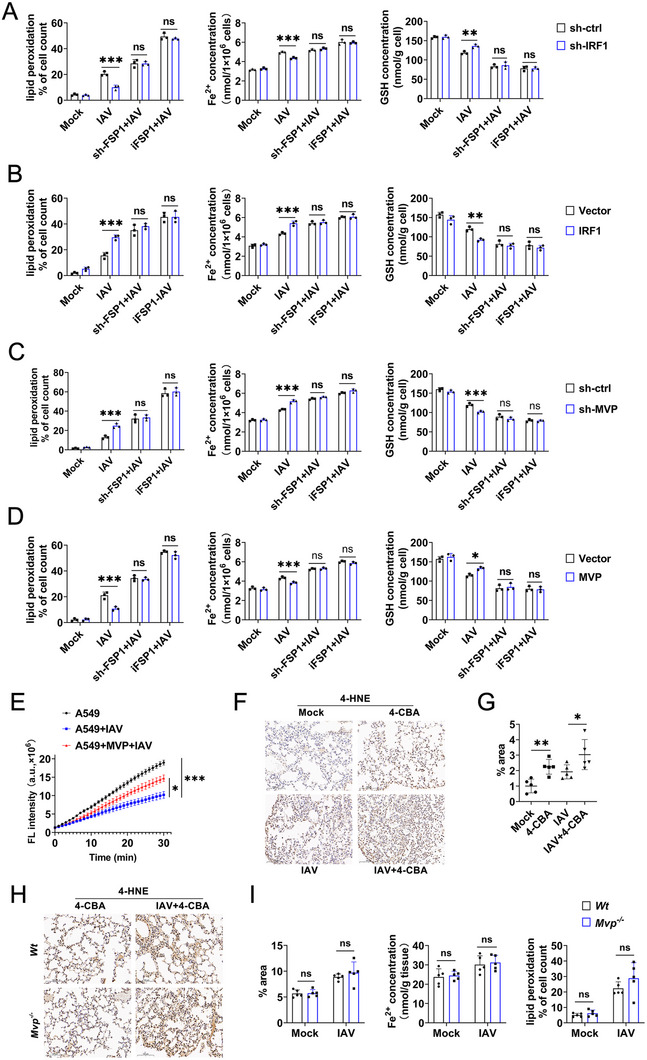
MVP and IRF1 regulated IAV‐induced ferroptosis via FSP1. (A) A549 cells were transfected with the indicated shRNAs for 36 h and treated with or without iFSP1 (10 µm) for 12 h. Then, cells were infected with or without IAV (MOI = 0.1) for 12 h, and lipid peroxidation levels, Fe^2+^ concentrations, and GSH levels were measured. (B) Experiments were similar to those in (A), except cells were transfected with vector control or pCMV‐IRF1. (C,D) Experiments were similar to those in (A and B), except that cells were transfected with either sh‐MVP or pCMV‐MVP. (E) Representative reaction curves of FSP1 activity assay using immunopurified FSP1 protein from cells with the indicated treatment. (F,G) C57BL/6 mice were infected with IAV (1 × 10^4^ PFU) and/or intraperitoneally injected with 4‐CBA (50 mg/kg, once every 2 days) for 4 days, and representative histopathological changes in 4‐HNE‐stained lung tissues are shown (F). Graphs depict quantification of the 4‐HNE‐stained area (G). Scale bars: 100 µm. Error bars are means ± SD, *n* = 5 randomly selected magnification fields. (H) Experiments were similar to those in (F), except *Mvp^−/−^* mice were used. (I) Experiments were similar to those in (H), except that graphs depict quantification of the 4‐HNE‐stained area, Fe^2+^ concentration, and lipid peroxidation levels were measured. We acknowledge the use of GraphPad Prism 8.0, Adobe Photoshop CC2019, and SlideViewer 2.5 for generating this figure. All experiments were performed in triplicate. The data are presented as mean ± SD. Statistical significance was assessed using two‐way ANOVA analysis in (A–D and I), two‐tailed Student's *t*‐test in (E and G) for comparisons. *p < 0.05; **p < 0.01; ***p < 0.001. n.s. = not significant. See also Figure S7.

To verify that IRF1 regulates IAV‐induced ferroptosis via FSP1, we synthesized FSP1 shRNA and assessed its efficiency (Figure S7A,B). shRNA‐ FSP1#3 was selected for the knockdown of FSP1, as described below. FSP1 knockdown or FSP1 inhibitor (iFSP1) treatment abolished the IRF1 knockdown‐regulated levels of Fe^2+^, lipid peroxidation, and GSH during IAV infection (Figure 7A and Figure S7C,D). Similar results were obtained for the IRF1 overexpression system (Figure 7B and Figure S7E,F). Consistently, FSP1 knockdown or iFSP1 treatment abolished MVP knockdown or MVP overexpression, which regulated the levels of Fe^2+^, lipid peroxidation, and GSH during IAV infection (Figure 7C,D, and Figure S7G–J). Consistently, IAV reduced FSP1 oxidoreductase activity, but MVP overexpression restored it (Figure 7E).

Next, we investigated whether the MVP/IRF1 axis regulates IAV‐induced ferroptosis via FSP1 in vivo. Given that iFSP1 cannot be used for in vivo treatment, and that FSP1 and CoQ function in the same pathway to inhibit ferroptosis, we employed a CoQ biosynthesis inhibitor (4‐CBA) to inhibit FSP1 function. As shown in Figure 7F,G and Figure S7K,L, 4‐CBA treatment increased IAV‐induced levels of Fe^2+^ and 4‐HNE in lung tissues, as well as lipid peroxidation levels in PBMCs. Next, we verified whether MVP regulates IAV‐induced ferroptosis through FSP1 in vivo. As shown in Figure 7H,I, MVP knockout did not affect the IAV‐induced levels of Fe^2+^ or 4‐HNE in the lung tissues, nor did it alter the lipid peroxidation levels in the PBMCs of 4‐CBA‐treated mice. These findings suggest that FSP1 is a downstream signaling molecule in MVP/IRF1 axis‐regulated ferroptosis during IAV infection.

### 2.8 The MVP/IRF1/FSP1 Axis Directly Regulated IAV‐Induced Ferroptosis

Because MVP inhibits viral replication by inducing IFN, MVP may regulate IAV‐induced ferroptosis by affecting IAV replication. To address this question, we employed two approaches. The first is to investigate the role of the MVP in IAV‐induced ferroptosis in Vero cells deficient in type I IFNs due to genomic deletions [29]. Consistent with our previous results [22], overexpression or knockdown of MVP did not affect IAV replication in Vero cells (Figure S8A–F). The other is to adjust the IAV initial infection dose to ensure that the IAV replication level in MVP knockdown or overexpressed cells is the same as in control cells (referred to below as the adjust system). Results showed that the replication of IAV (infection dose, MOI = 0.09) in sh‐control‐transfected A549 cells is the same as the replication of IAV (infection dose, MOI = 0.05) in sh‐MVP‐transfected A549 cells (Figure S8G–J). Similarly, the replication of IAV (infection dose, MOI = 0.05) in control vector‐transfected A549 cells is the same as the replication of IAV (infection dose, MOI = 0.09) in pCMV‐MVP‐transfected A549 cells (Figure S8K–N). Importantly, MVP knockdown still increased IAV‐induced lipid peroxidation and Fe^2+^ accumulation, while decreasing IAV‐inhibited GSH levels and cell viability in Vero cells or in the adjusted system (Figure S8O,P). By contrast, MVP overexpression inhibited IAV‐induced lipid peroxidation and Fe^2+^ accumulation, while enhancing IAV‐inhibited GSH levels and cell viability in Vero cells or in the adjusted system (Figure S8Q,R).

Next, we investigate whether IRF1/FSP1 regulates IAV‐induced ferroptosis by affecting IAV replication. As shown in Figure S9A–I, IRF1 knockdown, but not FSP1 knockdown or 4‐CBA treatment, promotes the replication of IAV. Thus, we select IRF1 for further research. The replication of IAV (infection dose, MOI = 0.1) in sh‐control transfected A549 cells is the same as the replication of IAV (infection dose, MOI = 0.04) in sh‐IRF1 transfected A549 cells (Figure S9G–M). Similarly, the replication of IAV (infection dose, MOI = 0.02) in control vector‐transfected A549 cells is the same as the replication of IAV (infection dose, MOI = 0.1) in pCMV‐IRF1‐transfected A549 cells (Figure S9N–Q). Of note, IRF1 knockdown decreased Fe^2+^ levels and lipid peroxidation, but increased GSH levels and cell viability during IAV infection in the adjusted system (Figure S9R). Similarly, IRF1 overexpression elevated Fe^2+^ and lipid peroxidation levels, but suppressed GSH levels and cell viability during IAV infection in the adjusted system (Figure S9S).

Finally, we investigate whether the MVP/IRF1 axis regulates IAV‐regulated FSP1 myristoylation by affecting IAV replication. Knockdown of IRF1 restores the inhibitory effect of IAV on FSP1 myristoylation, while overexpression of IRF1 enhances the inhibitory effect of IAV on FSP1 myristoylation in the adjusted system (Figure S10A–D). As expected, MVP knockdown synergistically inhibited IAV‐reduced FSP1 myristoylation; MVP overexpression recovered the FSP1 myristoylation inhibited by IAV in Vero cells or in the adjusted system (Figure S10E–L). These findings suggest that the MVP/IRF1/FSP1 axis directly regulates IAV‐induced ferroptosis, not by regulating IAV replication.

## 3 Discussion

**FIGURE 8:**
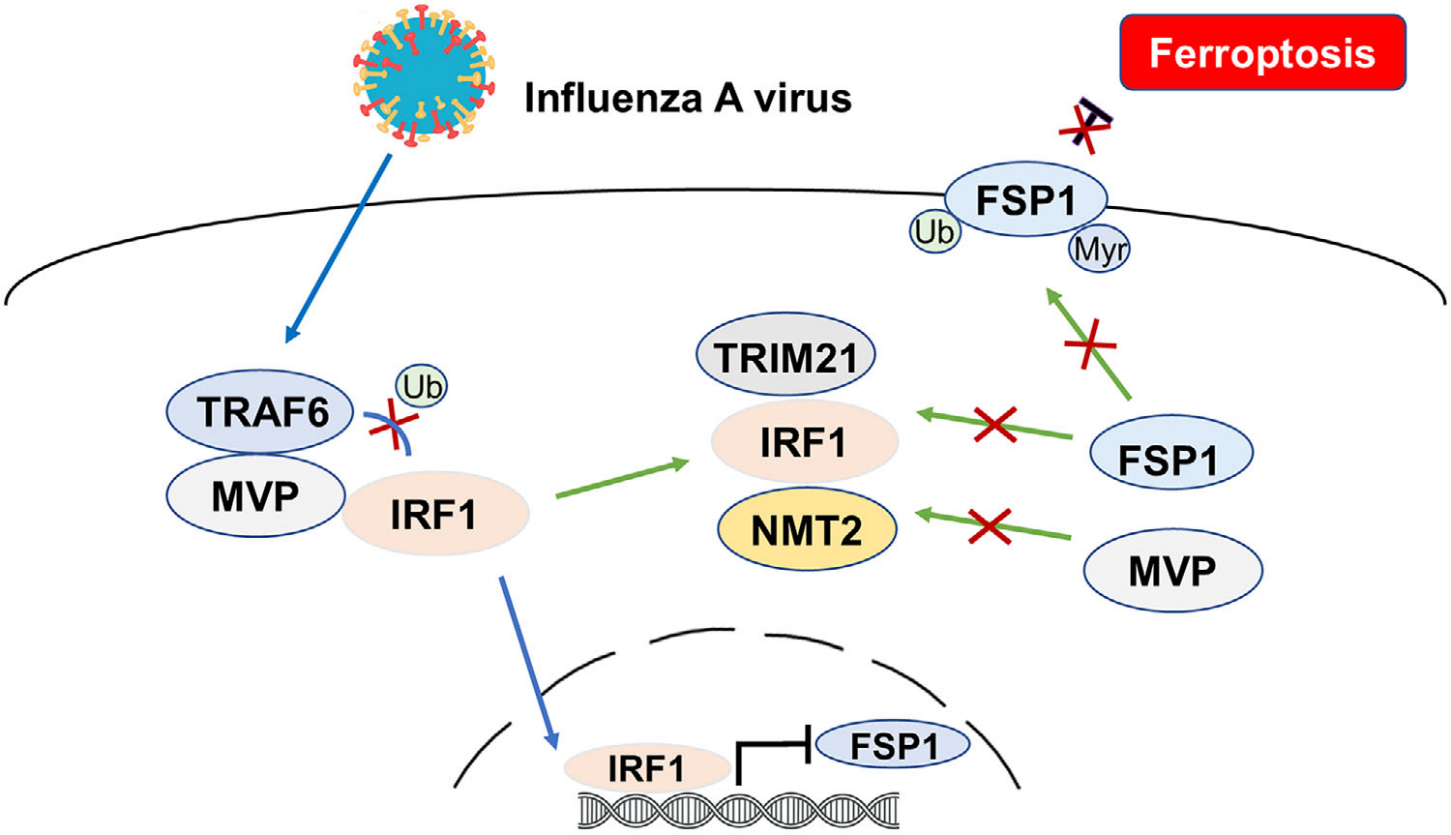
Working model depicts that IAV‐induced ferroptosis via the MVP‐IRF1‐FSP1 pathway. During IAV infection, MVP interacts with TRAF6 to destabilize its interaction with IRF1. As a result, non‐ubiquitinated IRF1 translocates to the cell nucleus, suppressing FSP1 at the transcription level (blue arrow). On the other hand, IAV infection induces interactions between IRF1 and NMT2 and between IRF1 and TRIM21, thereby releasing MVP and FSP1. As a result, non‐ubiquitinated and non‐myristoylated FSP1 do not translocate to the membrane, leading to ferroptosis.

Programmed cell death can be a manifestation of the “two sides of a coin” principle for host versus viral infection. On the one hand, the programmed cell death in an infected cell prevents the virus from completing its life cycle and producing new viruses. On the other hand, virus‐induced large‐scale cell death often leads to excessive organ damage. Thus, the homeostasis of programmed cell death is crucial for the “battle” between the host and viruses. In the present study, we identified two waves of regulated ferroptosis mediated by MVP in response to IAV infection (Figure 8). In the first wave, MVP associates with TRAF6, thereby disrupting IRF1‐TRAF6 complexes and IRF1 polyubiquitination. The released IRF1 translocates to the nucleus and binds to the promoter of FSP1, leading to the inactivation of FSP1 transcription (Figure 8). In the second wave, IRF1 associates with TRIM21 and NMT2, thereby preventing FSP1 from being ubiquitinated and myristoylated. As a result, FSP1 cannot translocate to the membrane and functions as a ferroptosis suppressor (Figure 8). This unusual and intriguing phenomenon raises the question of why host cells employ two waves of MVP‐mediated ferroptosis suppression. To the best of our knowledge, this is a dual insurance strategy for the host to ensure the effectiveness of the MVP‐FSP1 axis, which is crucial for combating IAV infection.

Two studies have shown that IAV infection regulates ferroptosis. One study has shown that in nasal mucosal epithelial cells, IAV infection induced ferroptosis via the NRF2‐KEAP1‐GCLC pathway [30]. The other study reported that IAV hemagglutinin interacts with autophagic receptors NCOA4 and TAX1BP1 to facilitate ferritinophagy [14]. However, little is known about the mechanism of ferroptosis surveillance during IAV infection. There are two central mechanisms for suppressing ferroptosis. GPX4 mediates the reduction of phospholipid peroxides. Another type is enzyme‐mediated, such as FSP1, which produces metabolites with free radical‐scavenging antioxidant activity. In this study, we found that the MVP‐IRF1 axis is a type of ferroptosis surveillance that is dependent on FSP1 expression. Notably, we found no significant change in GPX4 expression in IAV‐infected cells. In addition, analysis of clinical samples revealed that GPX4 does not participate in IAV‐regulated ferroptosis, suggesting that the host selects FSP1 explicitly for ferroptosis surveillance during IAV infection.

The location and post‐translational modification of FSP1 are compelling. Increasing evidence indicates that FSP1 translocation to the plasma membrane is an essential step for its inhibitory effect on ferroptosis. In addition, FSP1 can be ubiquitinated or myristoylated. However, the relationship between these two types of post‐translational modifications of FSP1 has not yet been investigated. In this study, we showed that FSP1 myristoylation and K63‐linked ubiquitination contributed to its plasma membrane localization. Studies have demonstrated that blocking FSP1 ubiquitination inhibits its myristoylation. In contrast, the suppression of FSP1 myristoylation did not affect its ubiquitination. These data indicated that induced FSP1 ubiquitination mediated by TRIM21 is an upstream step in FSP1 myristoylation.

MVP is a significant component of the vault protein complex. MVP is involved in various cellular processes, including apoptosis, autophagy, and multidrug resistance [18, 31, 32]. Our previous studies have shown that various viral infections, including IAV, hepatitis B virus, hepatitis C virus, and enterovirus 71, induce elevated MVP expression [22]. Mechanistic studies have shown that MVP interacts with MyD88 and other transcription factors, resulting in IFN expression and the release of pro‐inflammatory cytokines. Our previous studies have shown that MVP is a crucial protein in innate immunity and inflammatory response. In this study, we demonstrated that MVP inhibited IAV‐induced ferroptosis by competitively inhibiting IRF1‐mediated ferroptosis. Given our results, we propose that the MVP is a “key node” in host ferroptosis and innate immunity against viral infection. In addition, we observed decreased FSP1 expression, which correlated with increased MVP expression in patients with IAV (Figure 1), suggesting that the ferroptosis‐suppressing function of MVP was impaired, whereas its pro‐inflammatory function was retained.

It has yet to slip our attention that there are several important caveats. (i) MVP is the major component of the vault complex. We did not investigate whether free MVP or the vault component of MVP regulated IAV‐induced ferroptosis. (ii) Why did the host choose MVP‐IRF1‐FSP1, but not GPX4, as a major surveillance mechanism? (iii) FSP1 is a critical component of a nonmitochondrial coenzyme Q_10_ (CoQ) antioxidant system. Would CoQ influence antiviral response in patients with IAV? Studies exploring these questions would help clarify how programmed cell death regulates viral infection. Although further studies are needed to address these limitations, our findings reveal a novel ferroptosis surveillance mechanism during viral infection.

## 4 Methods

### 4.1 Ethics Statement

The collection of clinical samples was performed in accordance with the principles of the Declaration of Helsinki and also authorized by the Medical Ethics Committee of Wuhan University (Approval No. WHU‐LFMD‐2023051), following guidelines for the protection of human subjects. All research participants provided written informed consent for the collection of specimens and follow‐up analyses.

All animal experiments were conducted following the Chinese National Laboratory Animal Guideline for Ethical Review of Animal Welfare. The animal protocols were approved by the Institutional Animal Care and Use Committee of Wuhan University (Approval No. WDSKY0201302).

### 4.2 Clinical Specimens

All clinical samples were obtained from Zhongnan Hospital of Wuhan University (Wuhan, Hubei, China). Obtain blood samples from patients diagnosed with H1N1 influenza virus infection. Randomly select HI blood samples from the Medical Examination Center as controls. In this study, influenza patients were confirmed to be infected with IAV by quantitative PCR (qPCR). Separate PBMCs from peripheral blood using Ficoll gradient centrifugation.

Please refer to Tables S1 and S2 for detailed information on the samples.

### 4.3 Cell Culture, Virus, and Reagents

All human cell lines, A549 (RRID: CVCL_0023), Vero (RRID: CVCL_0059), and HEK293T (RRID: CVCL_0063), were purchased from the American Type Culture Collection. Cell line identities were verified by commercial short tandem repeat profiling with database matching, confirming the absence of cross‐contamination. A549 cultured in F‐12K medium and Vero and HEK293T cultured in Dulbecco's modified Eagle's medium (DMEM), cell culture medium added 10% fetal bovine serum (FBS), 100 U/mL streptomycin‐penicillin. All cells were incubated in a 37°C, 5% CO_2_ incubator. All cells were tested monthly for mycoplasma contamination using the One‐Step Mycoplasma Detection Kit (Yeasen Biotech, Shanghai, China). Table S3 provides detailed information on cell cultures, antibodies, and other reagents used in this study.

The recombinant human IAV A/WSN/33 (H1N1) virus was transfected into Madin‐Darby canine kidney cells using an eight‐plasmid transfection system to produce IAV [33]. This eight‐plasmid transfection system was a gift from Professor R.G. Webster (Department of Infectious Diseases, St. Jude's Children's Research Hospital, Memphis, TN, USA).

### 4.4 Plasmids

Flag‐FSP1, Flag‐MVP, Flag‐TRAF6, HA‐MVP, and HA‐IRF1 were constructed by cloning corresponding cDNA into the pCMV‐Flag or pCMV‐HA vector, respectively. FSP1‐eGFP, MVP‐eGFP, IRF1‐eGFP, TRAF6‐eGFP, FSP1‐mCherry, MVP‐mCherry, and IRF1‐mCherry were generated by insertion of the corresponding coding sequence into pcDNA3.1‐GFP or pcDNA3.1‐mCherry. Insert the Lyn11 sequence (MGCIKSKGKDS) and a linker into pcDNA3.1‐mCherry to construct Lyn11‐mCherry. This sequence encodes a peptide that targets proteins to the plasma membrane. All plasmids we constructed were confirmed by DNA sequencing detection (Tsingke, Beijing, China). To confirm the specificity of the constructs and antibodies, all constructed plasmids were transfected into 293T cells and tested for plasmid expression by Western blot. The shRNAs in this study are shown in Table S4. Sigma–Aldrich Corporation has tested the knockdown efficiency of these shRNAs, and we are still retesting their efficiency in the laboratory.

### 4.5 Transgenic Mice

MVP‐knockout C57BL/6 (*Mvp^−/−^* mice) was a gift from Professor Erik A.C. Wiemer (Erasmus MC Cancer Institute, University Medical Center Rotterdam) [24]. Wild‐type C57BL/6 (*Wt*) mice were purchased from the Centers for Disease Control and Prevention (Wuhan, Hubei, China). All mice in this study were kept under an SPF environment. During the experiment, when the mice meet certain clinical standards, or the experiment is finished, they will be euthanized under humane conditions.

### 4.6 Real‐Time PCR

Using TRIzol reagent (TAKARA) to extract total RNA. Using the Bio‐Rad T100 real‐time PCR system, perform the quantitative PCR assays using the SYBR‐GREEN One‐Step kits (Bio‐Rad). Specific primers were listed in Table S5. Using the 2^−ΔΔCt^ method to calculate and normalize the relative expression of each gene.

### 4.7 Western Blot Analysis and Co‐Immunoprecipitation

Cells were washed and lysed in RIPA buffer. Protein concentrations in the lysate were measured using a BCA assay (Bio‐Rad). Separate 40 mg of protein from each sample using SDS‐PAGE, then transfer it onto a nitrocellulose membrane (Bio‐Rad). Seal the membrane using 5% (w/v) skim milk at 37°C for 1 h. Then, incubate the membrane with the primary antibody at 4°C for 12 h. Subsequently, the imprint was incubated with a secondary antibody conjugated to horseradish peroxidase for 1 additional hour. Observation of immune response bands using a chemiluminescence system (GE Healthcare).

For Co‐IP, cells were harvested and then lysed in NP‐40 buffer (20 mm Tris‐HCl (pH 7.4), 150 mm NaCl, 1% protease inhibitors, and 1% NP‐40). Then, add 20 µL protein A/G agarose to the lysis buffer for 2 h of pre‐washing, and add an appropriate amount of IgG or designated antibody to the supernatant at 4°C for 12 h. Then, target proteins in the lysate were captured by protein A/G beads. The binding beads were washed seven times with a washing buffer containing 1% NP‐40 and 1 M NaCl. Total protein and Co‐IP samples were processed according to the Western blot protocol using the indicated antibodies.

### 4.8 Cell Death and Viability Assays

Inoculate the cells into a 12‐ or 96‐well plate. After 12 h, PI staining and flow cytometry are used to measure cell death after the relevant treatment. At room temperature, incubate cells with PI (2 µg/mL) for 30 min, then analyze using a Beckman CytoFLEX S (Beckman Coulter). Use the CCK‐8 method to determine cell survival rate. After appropriate treatment, the cells in the 96‐well plate were removed from the culture medium, washed with PBS, and then incubated in culture medium containing 10% CCK8 reagent. Incubate the mixture at 37°C for 2 h, then measure the OD at 450 nm to quantify cell viability.

### 4.9 Lipid Peroxidation Assay

Using BODIPY‐C11 dye to analyze the lipid peroxidation level of cells by flow cytometry. After seeding the cells in a 12‐well plate for 12 h, the cells were inoculated with IAV A/WSN/33 (H1N1) virus and treated with DMSO or Ferrostatin‐1 for 12 h. Then discard the original culture medium and add 1 milliliter of culture medium containing 10 µm BODIPY‐C11, and incubate in a cell incubator for 30 min. Wash the cells to remove excess BODIPY‐C11, resuspend the cells in PBS, filter the cell suspension using a cell filter, and then analyze by flow cytometry. Measure the cell fluorescence intensity using a Beckman Cyto FLECX S (Beckman Coulter) flow cytometer. Analyze at least 10 000 cells per sample.

### 4.10 Iron Measurement

Using the iron assay kit (ab83366) purchased from Abcam to measure intracellular ferrous (Fe^2+^) levels. Inoculate the cells onto a 10 cm cell culture dish, then infect with IAV A/WSN/33 (H1N1) virus and treat with DMSO or Ferrostatin‐1 for 12 h. Wash the cells and add five times the volume of the iron assay buffer. After homogenizing the cells on ice, centrifuge the homogenate at 12 000 × g, 4°C for 10 min, and collect the supernatant. Add the iron‐reducing agent, then incubate at room temperature for 30 min. Then add the iron probe; after thorough mixing, incubate at room temperature for 1 h. This process is placed in a dark place to avoid exposure to light. Measure the samples' absorbance at 593 nm.

### 4.11 GSH Measurement

GSH concentration was detected by Glutathione Assay Kit (CS0260; Sigma). Inoculate the cells into a 10 cm cell culture dish for 12 h, infect with IAV A/WSN/33 (H1N1), and treat with DMSO or Ferrostatin‐1 for 12 h. Then wash the cells and lyse them in 1% lysis buffer (300 mm NaCl, 25 mm Tris, 0.5% NP‐40, 1 mM EDTA, protease and phosphatase inhibitors). After ultrasonic treatment of the lysate, centrifuge at 12 000 rpm for 10 min at 4°C, and collect the supernatant to measure GSH concentration. Add the GSH measurement mixture to the samples and incubate at room temperature in the dark for 1 h. Measure the yellow product (5‐thio‐2‐nitrobenzoic acid) using spectrophotometry at 412 nm.

### 4.12 Luciferase Reporter Assay

293T cells were transfected with promoter constructs and the pRL‐TK luciferase reporter plasmid as an internal control. 48 h post‐transfection, remove the growth medium from the cells and wash twice with 1 × PBS. The dual luciferase reporter assay system (Promega, E1910) was used. Add 100 µL of 1× PLB buffer and shake for 30 min at room temperature to lyse. Then, collect the PLB lysate and add it to a 96‐well plate. Then add luciferase to each well and measure luciferase activity using a microplate reader.

### 4.13 Chromatin Immunoprecipitation (ChIP) Analysis

A final concentration of 1% formaldehyde was added to A549 cells at room temperature, crosslinked for 10 min, then a final concentration of 0.125 M glycine was added. Scrape off the cells with a scraper, then lyse them in cold lysis buffer (50 mm KCl, 10 mm Tris‐HCl, pH 8.0, 0.5% Triton X‐100, 5 mm EDTA, and protease inhibitor). After 10 min at 4°C, harvest the precipitate and resuspend it in ChIP buffer (50 mm Tris‐HCl, pH 8.0, 5 mM EDTA, 1% SDS, and protease inhibitor mixture) for lysis. After ultrasonic treatment, centrifuge at 4°C, 12 000 rpm, 10 min. Incubate the supernatant with the specific IRF1 antibody overnight, and add protein A/G beads for 2 h the next day. Wash the beads seven times with washing buffer (50 mm HEPES, pH 7.5, 500 mm NaCl, 1 mm EDTA, 0.7% sodium deoxycholate, and 1% NP‐40). Wash the protein DNA complex with 0.1 M NaHCO_3_ and 1% SDS, reverse crosslink overnight in a 55°C water bath, extract DNA, and perform quantitative PCR (qPCR).

### 4.14 Click‐It Chemistry

Add azide myristic acid to the corresponding treated cells up to 50 µm for 6 h to label the myristoylated protein. Collect cells and lyse them in RIPA buffer for 10 min, then centrifuge at 4°C and 12 000 rpm for 10 min to obtain the supernatant. Add the following reagents in sequence and mix well to prepare a click mixture: 5 µL of 20 mm biotin‐PEG4‐alkyne, 10 µL of 100 mm copper (II) sulfate, 10 µL of 100 mm tris (2‐carboxyethyl) phosphine (TCEP), and 5 µL of 20 mm tris (benzyltriazolyl) amine (TBTA). Then add this mixture to 170 µL of cell lysate. Then vortex the sample and incubate at room temperature for 60 min. Add 600 µL cold methanol to each sample, vortex, and store overnight at −80°C. Centrifuge the sample at 14 000 × g, 4°C, for up to 30 min to precipitate the sample protein. Wash the precipitate twice with 600 µL cold methanol, dry it, and resuspend it in PBS containing 1% SDS. Add 5 x loading buffer and analyze by SDS‐PAGE.

### 4.15 Immunofluorescence Microscopy

Cells grown on confocal dishes were fixed with 4% (w/v) paraformaldehyde, then treated with the 0.1% Triton X‐100 for 10 min. Use 5% BSA to block the sample at 37°C for 45 min, incubated with primary Abs overnight at 4°C, and then incubated with dye‐conjugated secondary Abs for 1 h at room temperature. The cells were then stained with 4′,6‐diamidino‐2‐phenylindole (DAPI) and DiIC18(3)(DiI). Use a confocal laser‐scanning microscope (Leica, Germany) to capture images.

### 4.16 Immunohistochemistry

Fix the tissue sections of the mouse model in 10% formalin and embed them in paraffin. The slices are deparaffinized in xylene, then rehydrated with a series of alcohols. Block endogenous peroxidase with 3% H_2_O_2_. Incubate the slices with the corresponding Abs overnight. Use the HRP‐DAB kit (ZLI‐9019, ZsBio) to detect antibody binding. Then counterstain the slices with hematoxylin. Replace specific antibodies with PBS for negative control samples. Obtain images using a Nikon microscope and Nikon imaging software.

### 4.17 FSP1 Enzyme Assay

Cells were harvested and then lysed in NP‐40 buffer (20 mm Tris‐HCl (pH 7.4), 150 mm NaCl, 1% protease inhibitors, and 1% NP‐40). Then, add 20 µL protein A/G agarose to the lysis buffer for 2 h of pre‐washing, and add FSP1 antibody to the supernatant at 4°C for 12 h. Then, the FSP1 protein in the lysate were captured by protein A/G agarose. The binding beads were washed by washing buffer containing 1% NP‐40 and 1 M NaCl. To elute the FSP1 protein bound to the beads, 0.1 M glycine‐HCl (pH 3.0) was added, and the mixture was incubated with rotation for 3 min. The glycine‐HCl eluate was immediately neutralized with 1 M Tris‐HCl (pH 8.0). The concentration of the eluted FSP1 protein was then determined. For the resazurin‐based FSP1 activity assay, reaction solutions in TBS buffer (50 mm Tris‐HCl, 150 mm NaCl) containing 10 ng isolated FSP1 immunopurified from A549 cells, 200 µm NADH were prepared. After the addition of 100 µm resazurin sodium salt, the fluorescence intensity (FL intensity, Ex/Em = 540 nm/590 nm) was monitored every 1 min at 37°C using SpectraMax iD5 microplate reader (Molecular devices).

### 4.18 Statistical Analysis

For the knockdown plasmid, three independent shRNAs are used to verify knockdown efficiency. Cell culture experiments are independently repeated at least three times, and the data are expressed as the mean ± SD, calculated from *n* = 3. For data normally distributed and having equal variances, statistical significance was determined by an unpaired two‐tailed Student's *t*‐test to compare two groups, and by two‐way ANOVA to compare multiple groups. Statistical significance (*p*‐value) was analyzed by GraphPad Prism 8.0 (GraphPad Software, San Diego, CA). *p*‐value < 0.05 is considered significant.

## Author Contributions

Y.C. and S.L. conceived and designed the experiment. Y.C., P.L., Y.X., and Z.L. performed the experiments. Y.C., Z.C., Q.Z., and S.W. analyzed the data. X.C., H.B., R.Q., and G.Z. processed and typeset the figures. Y.C., P.L., and S.L. wrote the manuscript. All authors read and approved the final manuscript.

## Funding

This work was supported by the National Key Research and Development Program of China (2023YFC2307800), the Natural Science Foundation of Hubei Province Innovation Group (2025AFA029), the Fundamental Research Funds for the Central Universities (2042024kf0011), the Natural Science Foundation of Wuhan (2024040701010031), the Fundamental Research Funds for the Central Universities (2042022dx0003), the National Natural Science Foundation of China (U22A20335).

## Conflicts of Interest

The authors declare no conflicts of interest.

## Supporting information




**Supporting File**: advs74657‐sup‐0001‐SuppMat.docx.

## Data Availability

All data needed to evaluate the conclusions in the paper are present in the paper and/or the Supplementary Materials. Additional data related to this paper may be requested from the authors.
