# Supplementary material for: MVP Inhibits Influenza A Virus‐Induced Ferroptosis by Targeting IRF1 and Increasing FSP1 Activity
Source: Adv Sci (Weinh). 2026 Mar 4;13(26):e20371. doi: 10.1002/advs.202520371 (PMC13159135; doi:10.1002/advs.202520371)
Supplement: Supplementary file 1 — Supporting File: advs74657‐sup‐0001‐SuppMat.docx. [file ADVS-13-e20371-s001.docx]

**Supporting Information**

**MVP Inhibits Influenza A Virus-Induced Ferroptosis by Targeting IRF1 and Increasing FSP1 Activity**

*Yingbo Chen, Paili Lin, Yongfang Xia, Zhiqiang Liu, Zilu Cheng, Qingmei Zhu, Shiqi Wan, Xiaoyu Chen, Haiyan Bao, Renbo Qiao, Gechang Zhong, YingZhu, Shi Liu^*^*

This file includes:

Figure S1 to S10

Table S1 to S5


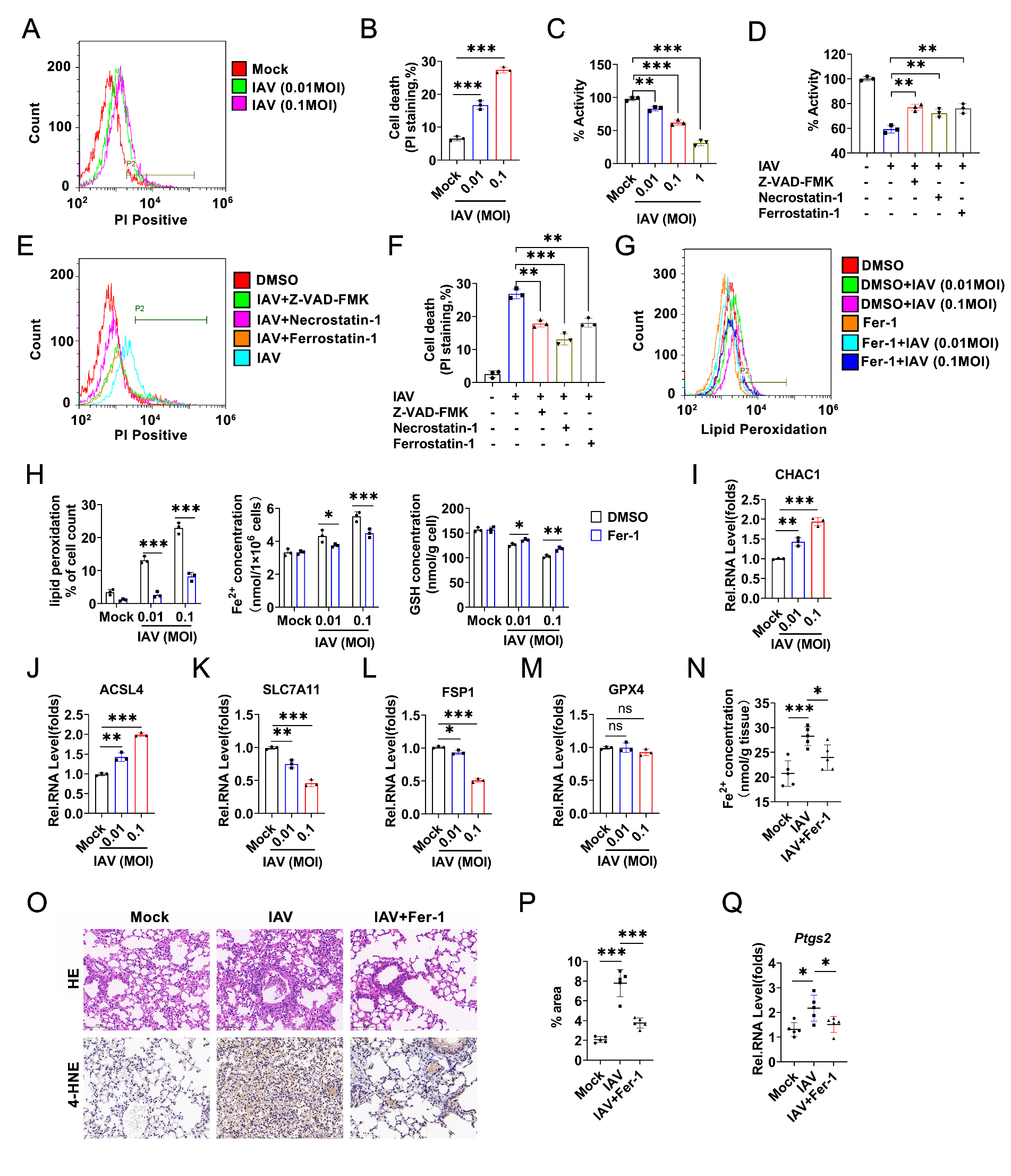


**Figure S1:** IAV infection induced ferroptosis *in vitro* and *in vivo* (related to Figure 1).

(A-C) A549 cells were infected with the indicated dose of IAV for 12 hrs, followed by flow cytometry analysis (A and B) or cell viability analysis (C).

(D-F) A549 cells were infected with IAV (MOI = 0.1) and treated with ferrostatin-1 (10 µM), Z-VAD-FMK (10 µM), or Necrostatin-1 (10 µM) for 12 hrs, followed by cell viability analysis (D) or flow cytometry analysis (E and F).

(G, H) A549 cells were infected with the indicated dose of IAV and treated with ferrostatin-1 (10 µM) for 12 hrs, followed by measuring lipid peroxidation levels, Fe^2+^ concentrations, and GSH levels.

(I-M) A549 cells were infected with the indicated dose of IAV for 12 hrs, followed by qPCR analysis.

(N) C57BL/6 mice were infected with IAV (1×10^4^ PFU) and/or intraperitoneally injected with ferrostatin-1 (10 mg/kg, twice a day) for 4 days, followed by measuring Fe^2+^ concentration in lung tissue. Error bars are means ± SD, n = 5.

(O, P) C57BL/6 mice were infected with IAV (1×10^4^ PFU) and/or intraperitoneally injected with ferrostatin-1 (10 mg/kg, twice a day) for 4 days. Representative histopathological changes in H&E-stained and 4-HNE-stained lung tissues (O). Graphs depict quantification of the 4-HNE-stained area (P). Scale bars: 100 μm. Error bars are means ± SD, n = 5 randomly selected magnification fields.

(Q) Experiments were performed similar to those in (N), except *Ptgs2* mRNA levels were analyzed.

We acknowledge the use of GraphPad Prism 8.0 (GraphPad Software, San Diego, CA), Adobe Photoshop CC2019 (Adobe Inc., San Jose, CA), SlideViewer 2.5 (3DHISTECH Ltd., Hungary) and CytExpert 2.4 (Beckman Coulter, Inc., USA) for generating this figure. All experiments were performed in triplicate. The data are presented as mean ± SD. Statistical significance was assessed using two-way ANOVA analysis in H, unpaired two-tailed Student's t-test in B-D, F, I-N, P and Q for comparisons. *p < 0.05; **p < 0.01; ***p < 0.001. n.s. = not significant.


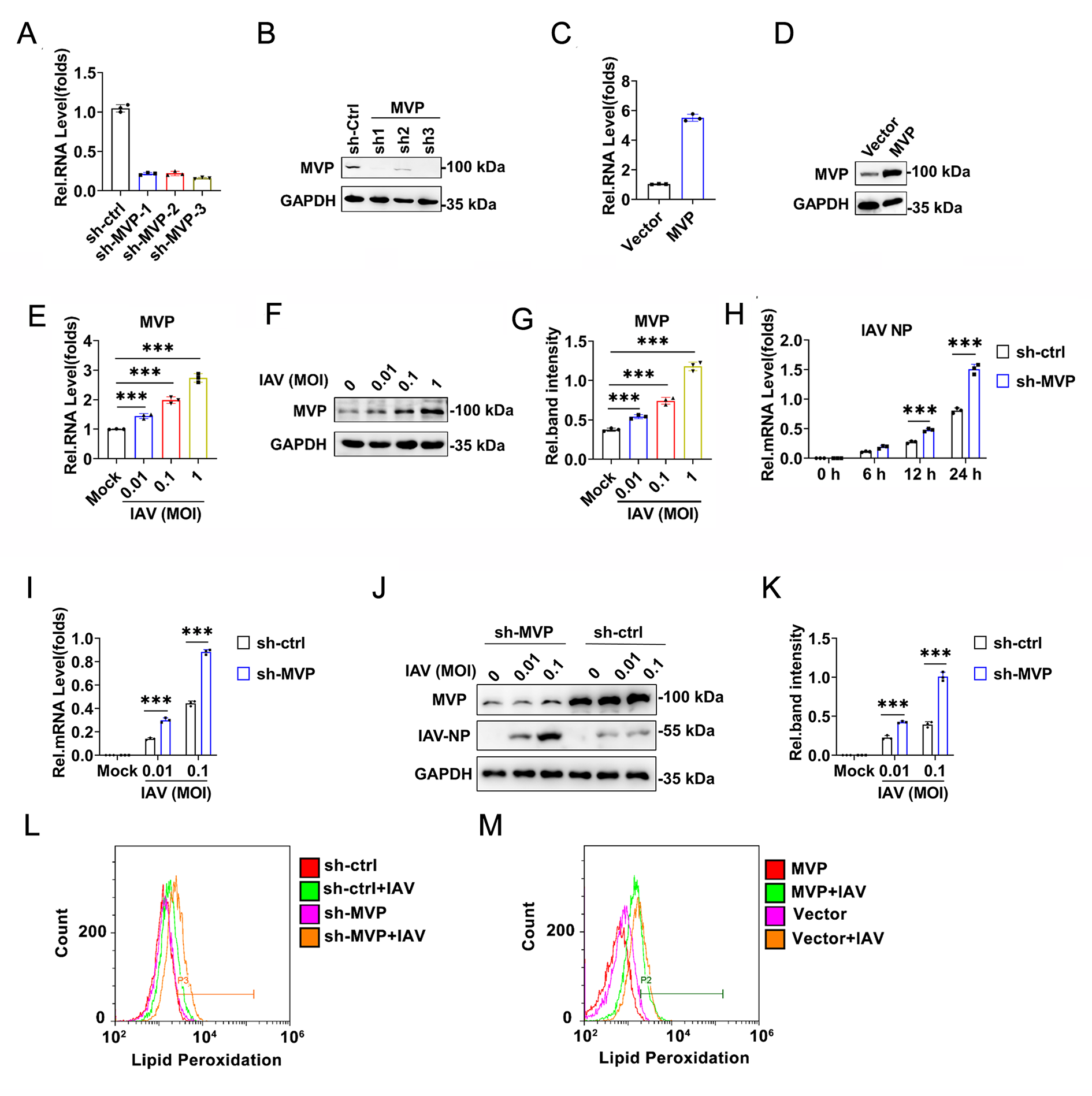


**Figure S2:** MVP inhibits IAV-induced ferroptosis *in vitro* and *in vivo* (related to Figure 2).

(A, B) A549 cells were transfected with the indicated shRNAs for 48 hrs before qPCR (A) and Western blotting (B) assays.

(C, D) Experiments were performed similarly to those in (A) and (B), except that cells were transfected with either a vector control or pCMV-MVP.

(E-G) A549 cells were infected with the indicated dose of IAV for 12 hrs before qPCR (E) and Western blotting (F) assays. The relative intensity of MVP was measured using ImageJ and normalized to its respective GAPDH (G).

(H) A549 cells were transfected with the indicated shRNAs, then infected with IAV (MOI = 0.1) for the indicated times, followed by measuring the RNA level of IAV nucleoprotein (NP).

(I-K) A549 cells were transfected with sh-ctrl or sh-MVP for 36 hrs and infected with IAV at the indicated dose for 12 hrs before qPCR (I) and Western blotting (J). The relative intensity of IAV NP was measured using ImageJ and normalized to their respective GAPDH (K).

(L) A549 cells were transfected with sh-control or sh-MVP for 36 hrs and infected with or without IAV (MOI = 0.1) for 12 hrs, followed by measuring lipid peroxidation levels.

(M) Experiments were performed similarly to those in (L), except cells were transfected with vector control or pCMV-MVP.

We acknowledge the use of GraphPad Prism 8.0, Adobe Photoshop CC2019 and CytExpert 2.4 for generating this figure. All experiments were performed in triplicate. The data are presented as mean ± SD. Statistical significance was assessed using two-way ANOVA analysis in H, I and K, two-tailed Student's t-test in E and G for comparisons. *p < 0.05; **p < 0.01; ***p < 0.001. n.s. = not significant.


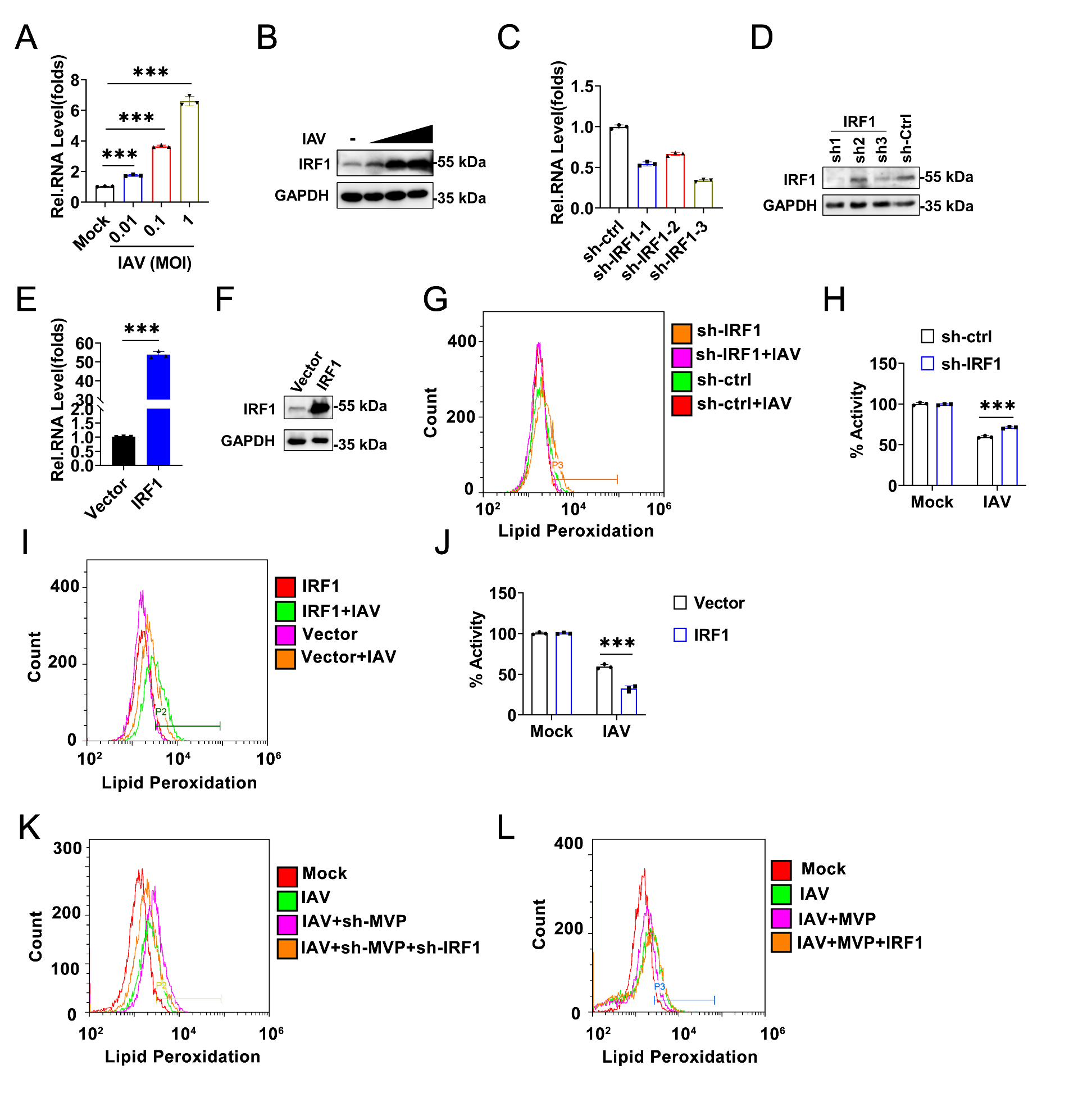


**Figure S3:** MVP associates with IRF1 to inhibit IAV-induced ferroptosis (related to Figure 3).

(A, B) A549 cells were infected with IAV (MOI = 0.1) for 12 hrs and subjected to qPCR (A) and Western blotting (B) assays.

(C, D) A549 cells were transfected with the indicated shRNAs for 48 hrs before qPCR (C) and Western blotting (D) assays.

(E, F) Experiments were performed similarly to those in (C and D), except that cells were transfected with either a vector control or pCMV-IRF1.

(G, H) A549 cells were transfected with sh-ctrl or sh-IRF1 for 36 hrs and infected with or without IAV (MOI = 0.1) for 12 hrs, followed by measuring lipid peroxidation levels (G) and cell viability (H).

(I, J) Experiments were performed similarly to those in (G and H), except that cells were transfected with either a vector control or pCMV-IRF1.

(K) A549 cells were transfected with the indicated shRNAs for 36 hrs and infected with or without IAV (MOI = 0.1) for 12 hrs, followed by measuring lipid peroxidation levels.

(L) Experiments were performed similarly to those in (K), except cells were transfected with the indicated plasmids.

We acknowledge the use of GraphPad Prism 8.0, Adobe Photoshop CC2019 and CytExpert 2.4 for generating this figure. All experiments were performed in triplicate. The data are presented as mean ± SD. Statistical significance was assessed using two-way ANOVA analysis in H and J, two-tailed Student's t-test in A for comparisons. *p < 0.05; **p < 0.01; ***p < 0.001. n.s. = not significant.


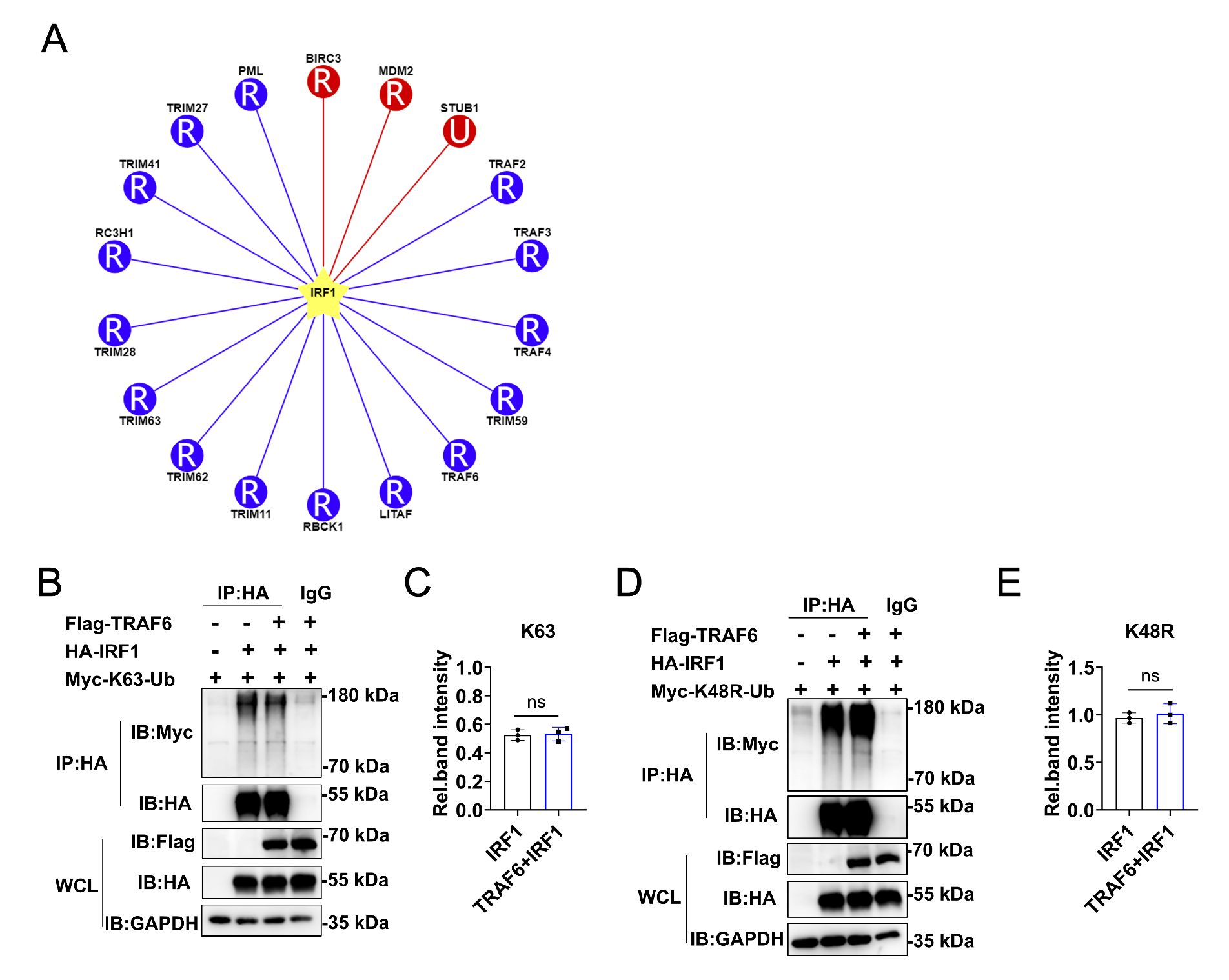


**Figure S4:** MVP inhibits K48-linked polyubiquitination of IRF1 via competitive binding to TRAF6 (related to Figure 4).

(A) UbiBrowser predicts the E3 ubiquitin ligase interacting with IRF1.

(B, C) 293T cells were transfected with the indicated plasmids for 36 hrs, then treated with MG132 for 4 hours. Co-IP and immunoblot analyses were performed with the indicated antibodies (B). The relative intensity of K63-linked polyubiquitination of IRF1 was measured using ImageJ and normalized to the respective unmodified IRF1 (C).

(D, E) Experiments were similar to those in (B and C), except that Myc-K48R-Ub was used.

We acknowledge the use of GraphPad Prism 8.0 and Adobe Photoshop CC2019 for generating this figure. All experiments were performed in triplicate. The data are presented as mean ± SD. Statistical significance was assessed using two-tailed Student's t-test for comparisons. *p < 0.05; **p < 0.01; ***p < 0.001. n.s. = not significant.


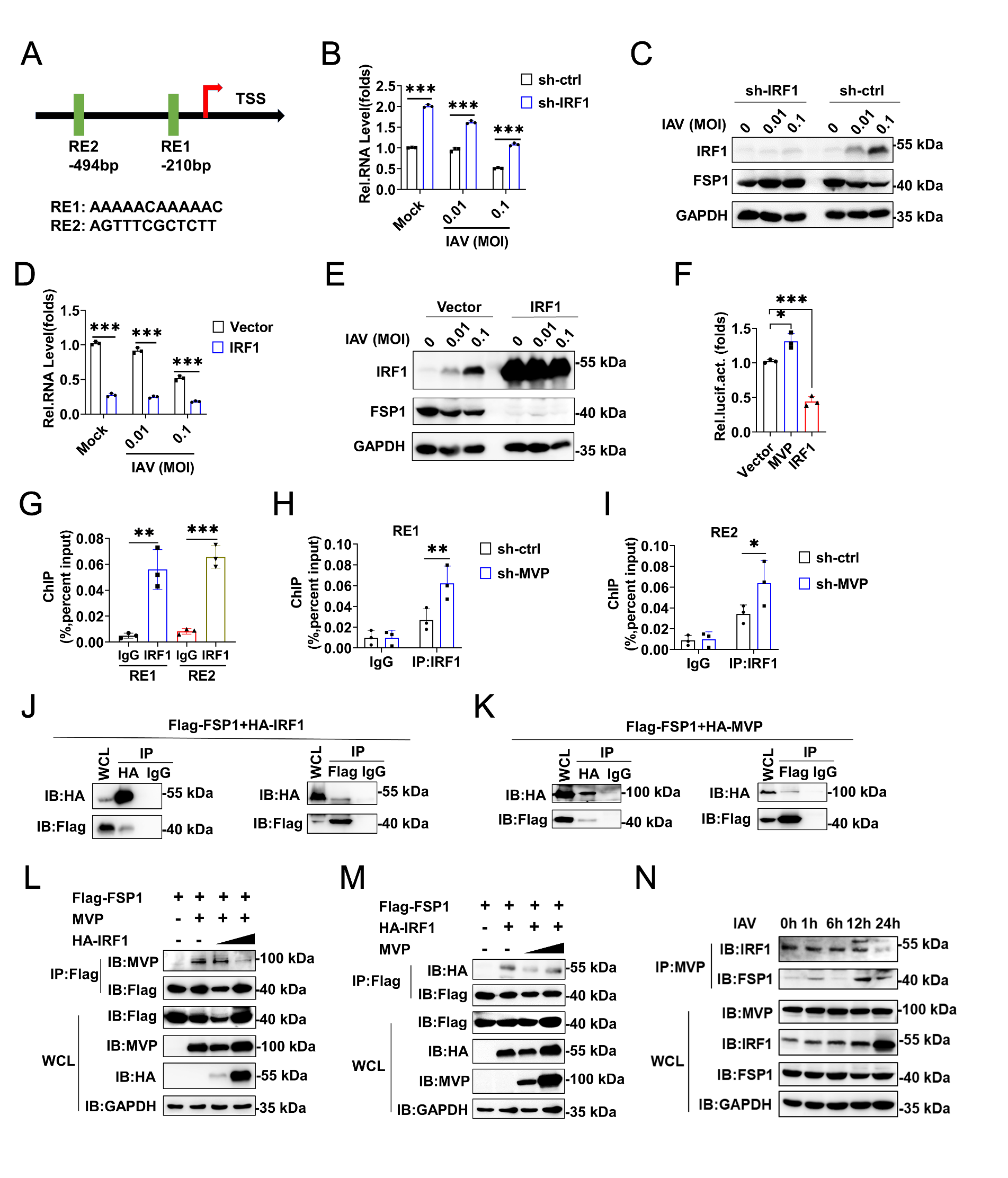


**Figure S5:** The MVP/IRF1 axis regulates the expression and localization of FSP1 during IAV infection (related to Figure 5).

(A) Schematic representation of the promoter region in the human FSP1 gene. The IRF1-binding sites upstream of the first exon are indicated as responsive elements. TSS, transcription start site.

(B, C) A549 cells were transfected with shRNA-control or shRNA-IRF1 for 36 hrs and infected with or without the indicated dose of IAV for 12 hrs and subjected to qPCR (B) and Western blotting (C) assays.

(D, E) Experiments were performed similarly to those in (B and C), except that cells were transfected with either a vector control or pCMV-IRF1.

(F) 293T cells were transfected with vector control, pCMV-IRF1, pCMV-MVP/or FSP1-luc for 36 hrs prior to luciferase activity reporter assays.

(G) ChIP-qPCR analyses of IRF1 binding on ARE1 and ARE2 in the FSP1 promoter in A549 cells.

(H, I) A549 cells were transfected with shRNA-control or shRNA-MVP for 48 hrs. ChIP assays were performed with anti-IRF1- or IgG-conjugated agarose. Promoter sequences in the input DNA and the DNA recovered from antibody-bound chromatin segments were detected using qPCR.

(J) 293T cells were transfected with Flag-FSP1 and HA-IRF1 for 48 hrs. Co-IP and immunoblot analyses were performed with the indicated antibodies.

(K) Experiments were performed similar to those in (J), except that HA-MVP was used.

(L) 293T cells were transfected with Flag-FSP1 (2 μg), MVP (2μg), and increasing amounts of HA-IRF1 (0.5, 2 μg) expression plasmids for 48 hrs. Co-IP and immunoblot analyses were performed with the indicated antibodies.

(M) 293T cells were transfected with Flag-FSP1 (2 μg), HA-IRF1 (2μg), and increasing amounts of MVP (0.5, 2 μg) expression plasmids for 48 hrs. Co-IP and immunoblot analyses were performed with the indicated antibodies.

(N) A549 cells were infected with IAV (MOI = 0.1) at the indicated times, underwent Co-IP and immunoblot analyses using the indicated antibodies.

We acknowledge the use of GraphPad Prism 8.0 and Adobe Photoshop CC2019 for generating this figure. All experiments were performed in triplicate. The data are presented as mean ± SD. Statistical significance was assessed using two-way ANOVA analysis in B, D, G-I, two-tailed Student's t-test in F for comparisons. *p < 0.05; **p < 0.01; ***p < 0.001. n.s. = not significant.


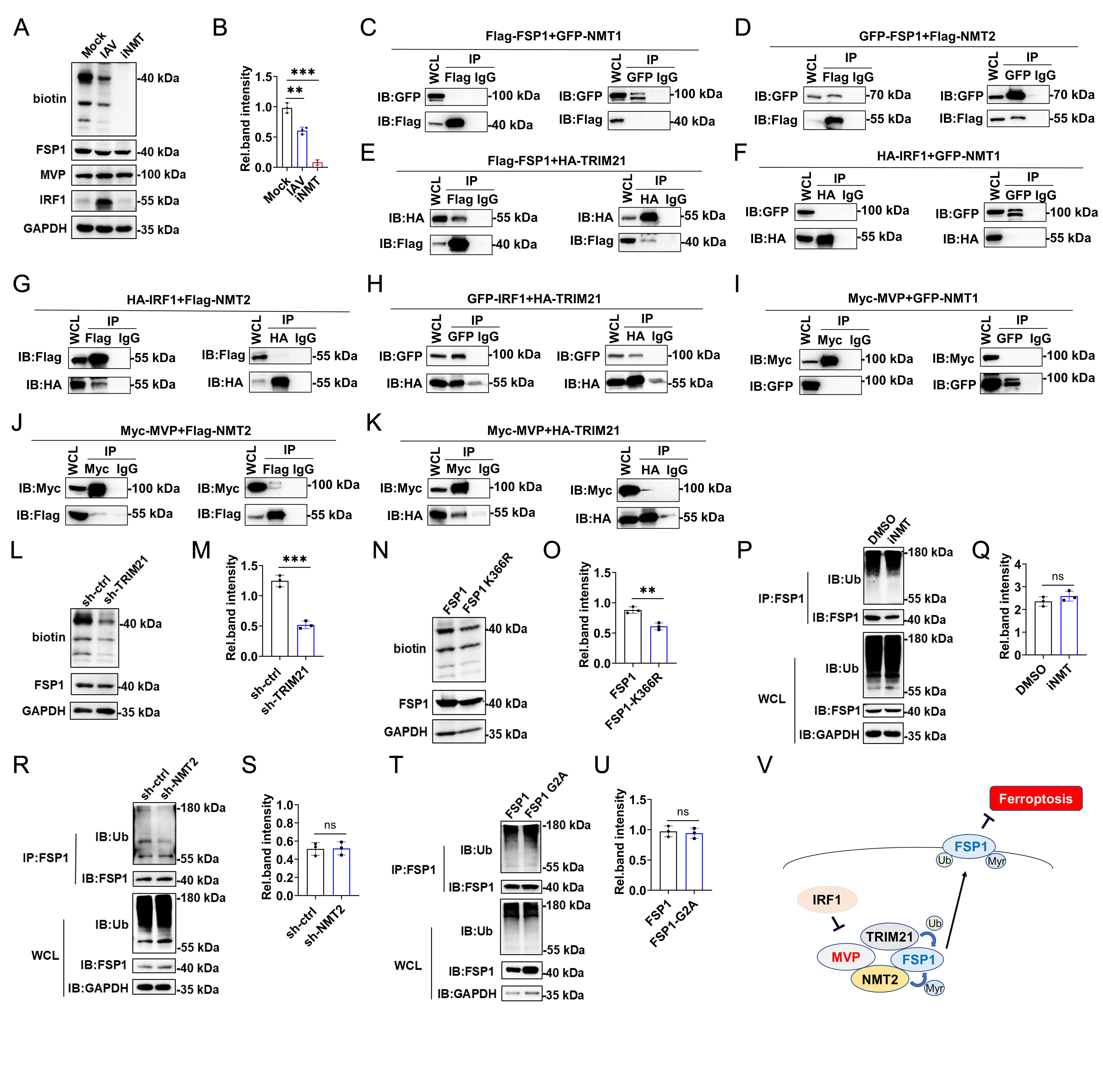


**Figure S6:** MVP/IRF1 axis regulated the ubiquitination and myristoylation of FSP1 (related to Figure 6).

(A, B) A549 cells were infected with or without IAV (MOI = 0.1) or treated with iNMT (10 µM) for 12 hrs, followed by click chemistry analyses (A). The relative intensity of myristoylation of FSP1 was measured using ImageJ and normalized to the respective unmodified FSP1 (B).

(C-K) 293T cells were transfected with the indicated plasmid for 48 hrs. Co-IP and immunoblot analyses were performed with the indicated antibodies.

(L-O) A549 cells were transfected with the indicated plasmids or shRNAs for 48 hrs, followed by click chemistry analyses. The quantification of the Western blots is shown in the adjacent graphs.

(P, Q) A549 cells were treated with iNMT (10 µM) for 12 hrs. Co-IP and immunoblot analyses were performed with the indicated antibodies (P). The relative intensity of polyubiquitination of FSP1 was measured using ImageJ and normalized to the respective unmodified FSP1 (Q).

(R, S) A549 cells were transfected with sh-control or sh-NMT2 for 48 hrs. Co-IP and immunoblot analyses were performed with the indicated antibodies (R). The relative intensity of polyubiquitination of FSP1 was measured using ImageJ and normalized to the respective unmodified FSP1(S).

T, U) 293T cells were transfected with pCMV-FSP1 or pCMV-FSP1-G2A for 48 hrs. Co-IP and immunoblot analyses were performed with the indicated antibodies (T). The relative intensity of polyubiquitination of FSP1 was measured using ImageJ and normalized to the respective unmodified FSP1 (U).

V) Schematic depicting MVP and IRF1 regulating the activity of FSP1. By IRF1 and FSP1 interactions, MVP recruits FSP1, NMT2, and TRIM21 to form a quaternary complex. As a result, the myristoylated and ubiquitinated FSP1 move to the plasma membrane and act as a ferroptosis suppressor.

We acknowledge the use of GraphPad Prism 8.0 and Adobe Photoshop CC2019 for generating this figure. All experiments were performed in triplicate. The data are presented as mean ± SD. Statistical significance was assessed using two-tailed Student's t-test for comparisons. *p < 0.05; **p < 0.01; ***p < 0.001. n.s. = not significant.


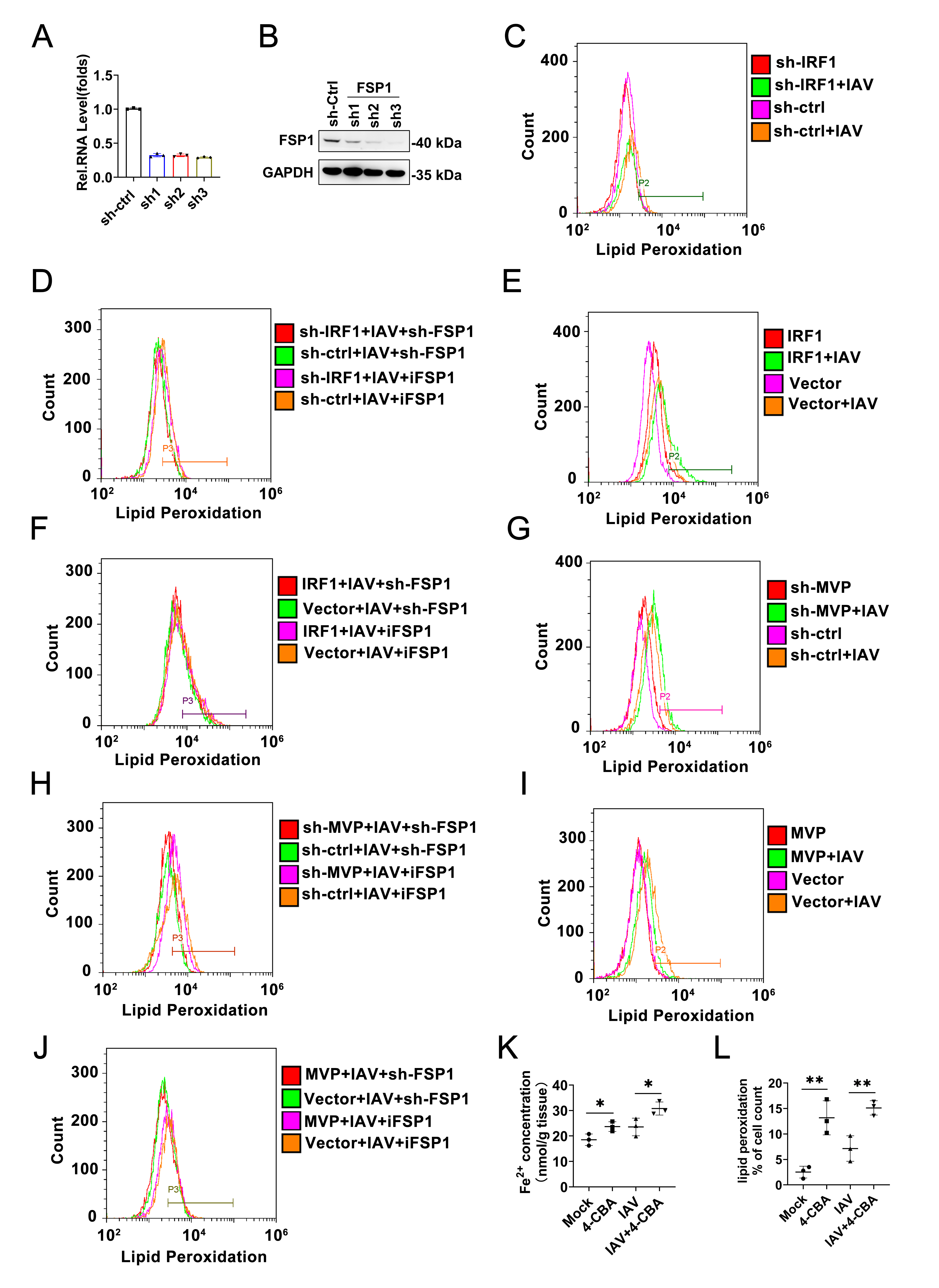


**Figure S7:** MVP/IRF1 axis-regulated IAV-induced ferroptosis via FSP1 (related to Figure 7).

(A, B) A549 cells were transfected with the indicated shRNAs for 48 hrs before qPCR (A) and western blotting (B) assays.

(C, D) A549 cells were transfected with the indicated shRNAs for 36 hrs and treated with or without iFSP1 (10 µM) for 12 hrs. Then, cells were infected with or without IAV (MOI = 0.1) for 12 hrs, followed by measurement of lipid peroxidation.

(E, F) Experiments were performed similarly to those in (C, D), except that cells were transfected with either a vector control or pCMV-IRF1.

(G-J) Experiments were performed similarly to those in (C-F), except cells were transfected with sh-MVP or pCMV-MVP.

(K, L) C57BL/6 mice were infected with IAV (1×10^4^ PFU) and/or intraperitoneally injected with 4-CBA (50mg/kg, once every 2 days) for 4 days, and Fe^2+^ concentration in lung tissues (K) and lipid peroxidation levels in PBMCs (L) were measured.

We acknowledge the use of GraphPad Prism 8.0, Adobe Photoshop CC2019 and CytExpert 2.4 for generating this figure. All experiments were performed in triplicate. The data are presented as mean ± SD. Statistical significance was assessed using two-tailed Student's t-test for comparisons. *p < 0.05; **p < 0.01; ***p < 0.001. n.s. = not significant.


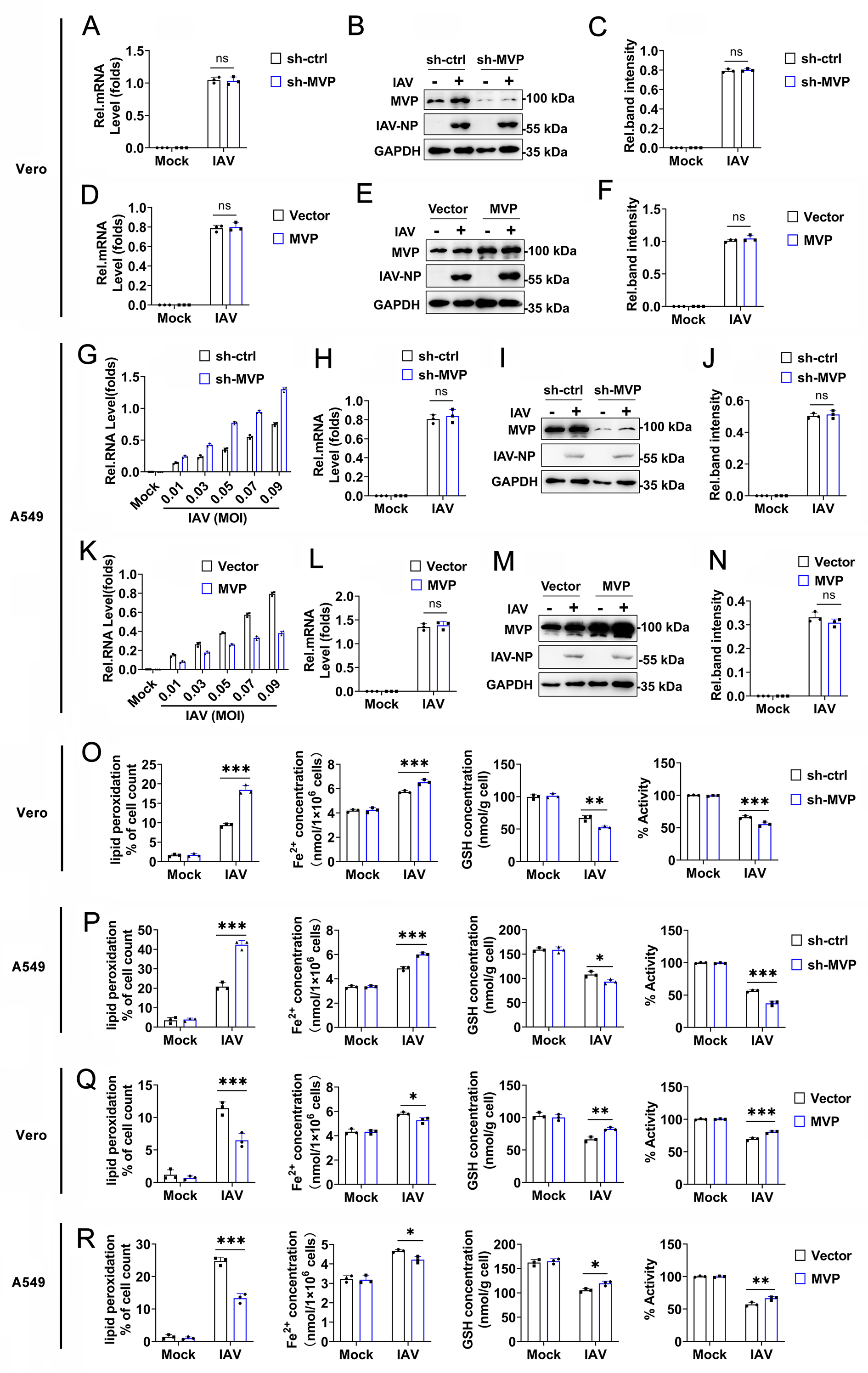


**Figure S8**: MVP directly regulated IAV-induced ferroptosis

(A-C) Vero cells were transfected with sh-ctrl or sh-MVP for 36 hrs and infected with or without IAV (MOI = 0.1) for 12 hrs before qPCR (A) and Western blotting (B) assays. The relative intensity of IAV NP was measured using ImageJ and normalized to their respective GAPDH (C).

(D-F) Experiments were similar to those in (A-C), except cells were transfected with vector control or pCMV-MVP.

(G) A549 cells were transfected with sh-ctrl or sh-MVP for 36 hrs and infected with indicated titers of IAV for 12 hrs, followed by measuring the RNA level of IAV NP.

(H-J) A549 cells were transfected with sh-ctrl or sh-MVP for 36 hrs. Then, sh-ctrl transfected cells were infected with IAV (MOI=0.09) for 12 hrs, and sh-MVP transfected cells were infected with IAV (MOI=0.05) for 12 hrs, and subjected to qPCR (H) and Western blotting (I) assays. The relative intensity of IAV NP was measured with the Image J program and normalized to their respective GAPDH (J)

(K) Experiments were similar to those in (G), except cells were transfected with vector control or pCMV-MVP.

(L-N) A549 cells were transfected with vector-ctrl or pCMV-MVP for 36 hrs. Then, vector-ctrl transfected cells were infected with IAV (MOI=0.05) for 12 hrs, and pCMV-MVP transfected cells were infected with IAV (MOI=0.09) for 12 hrs, and subjected to qPCR (L) and Western blotting (M) assays. The relative intensity of IAV NP was measured with the Image J program and normalized to their respective GAPDH (N)

(O) Vero cells were transfected with sh-control or sh-MVP for 36 hrs and infected with or without IAV (MOI = 0.1) for 12 hrs, followed by measuring lipid peroxidation levels, Fe^2+^ concentrations, GSH levels, and cell viability.

(P) Experiments were similar to those in (H-J), except that lipid peroxidation levels, Fe^2+^ concentrations, GSH levels, and cell viability were analyzed.

(Q) Experiments were similar to those in (O), except cells were transfected with vector control or pCMV-MVP.

(R) Experiments were similar to those in (L-N), except lipid peroxidation levels, Fe^2+^ concentrations, GSH levels, and cell viability were analyzed.

We acknowledge the use of GraphPad Prism 8.0 and Adobe Photoshop CC2019 for generating this figure. All experiments were performed in triplicate. The data are presented as mean ± SD. Statistical significance was assessed using a two-way ANOVA. *p < 0.05; **p < 0.01; ***p < 0.001. n.s. = not significant.


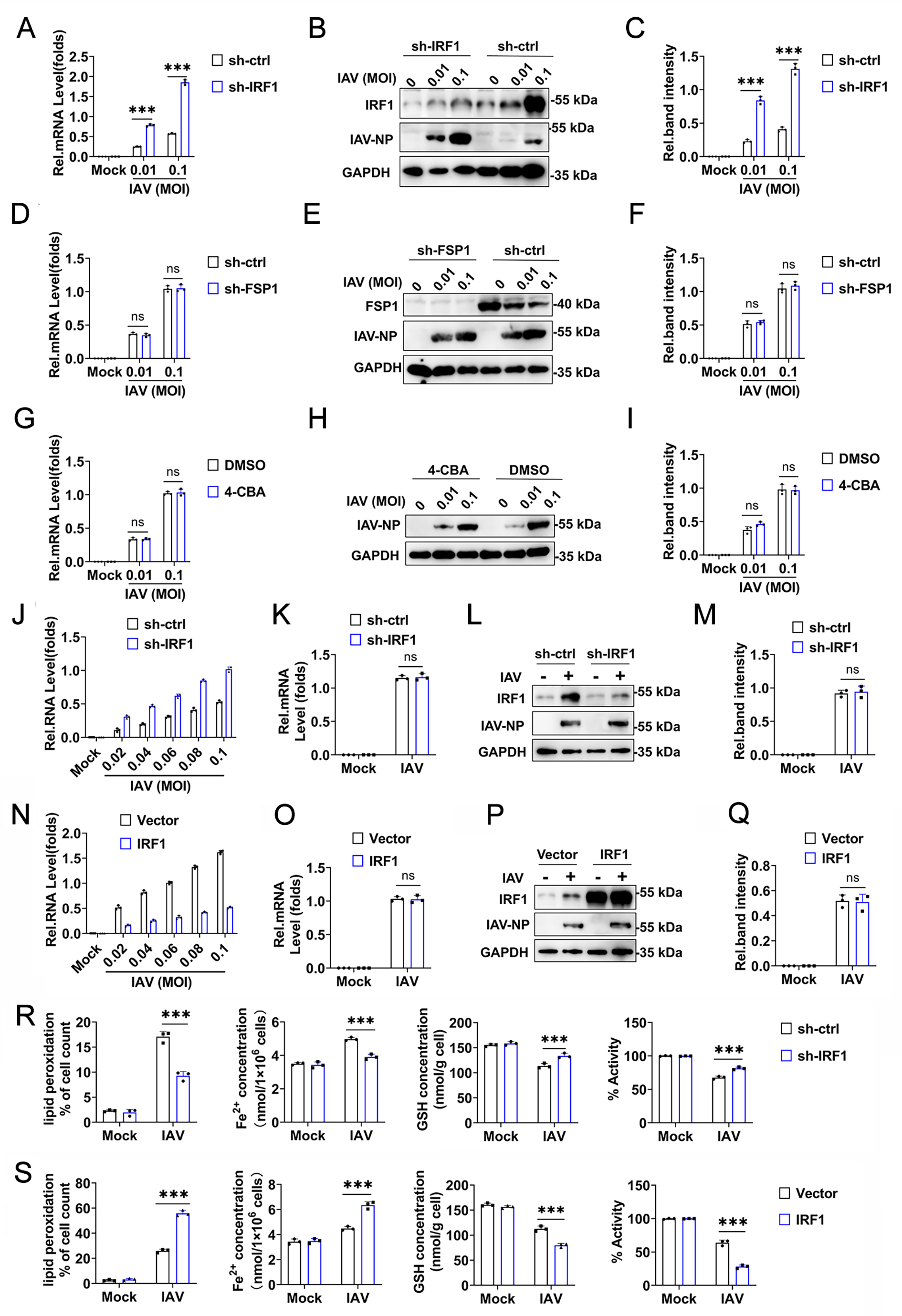


**Figure S9**: IRF1/FSP1 axis directly regulated IAV-induced ferroptosis

(A-C) A549 cells were transfected with sh-ctrl or sh-IRF1 for 36 hrs. Then, cells were infected with or without the indicated dose of IAV for 12 hrs before qPCR (A) and Western blotting (B) assays. The relative intensity of IAV NP was measured using ImageJ and normalized to their respective GAPDH (C).

(D-F) Experiments were similar to those in (A-C), except cells were transfected with sh-control or sh-FSP1.

(G-I) A549 cells were treated with 4-CBA (1 mM) or DMSO for 12 hrs before qPCR (G) and Western blotting (H). The relative intensity of IAV NP was measured using ImageJ and normalized to their respective GAPDH (I).

(J) A549 cells were transfected with sh-ctrl or sh-IRF1 for 36 hrs and infected with indicated titers of IAV for 12 hrs, followed by measuring the RNA level of IAV NP.

(K-M) A549 cells were transfected with sh-ctrl or sh-IRF1 for 36 hrs. Then, sh-ctrl transfected cells were infected with IAV (MOI=0.1) and sh-IRF1 transfected cells were infected with IAV (MOI=0.04) for 12 hrs, and subjected to qPCR (K) and Western blotting (L) assays. The relative intensity of IAV NP was measured using ImageJ and normalized to their respective GAPDH (M).

(N) Experiments were similar to those in (J), except cells were transfected with vector control or pCMV-IRF1.

(O-Q) A549 cells were transfected with vector-ctrl or pCMV-IRF1 for 36 hrs. Then, vector-ctrl transfected cells were infected with IAV (MOI=0.02) and pCMV-IRF1 transfected cells were infected with IAV (MOI=0.1) for 12 hrs, and subjected to qPCR (O) and Western blotting (P) assays. The relative intensity of IAV NP was measured using ImageJ and normalized to their respective GAPDH (Q).

(R) A549 cells were similar to those in (K-M), except that lipid peroxidation levels, Fe^2+^ concentrations, GSH levels, and cell viability were analyzed.

(S) A549 cells were similar to those in (O-Q), except that lipid peroxidation levels, Fe^2+^ concentrations, GSH levels, and cell viability were analyzed.

We acknowledge the use of GraphPad Prism 8.0 and Adobe Photoshop CC2019 for generating this figure. All experiments were performed in triplicate. The data are presented as mean ± SD. Statistical significance was assessed using a two-way ANOVA. *p < 0.05; **p < 0.01; ***p < 0.001. n.s. = not significant.


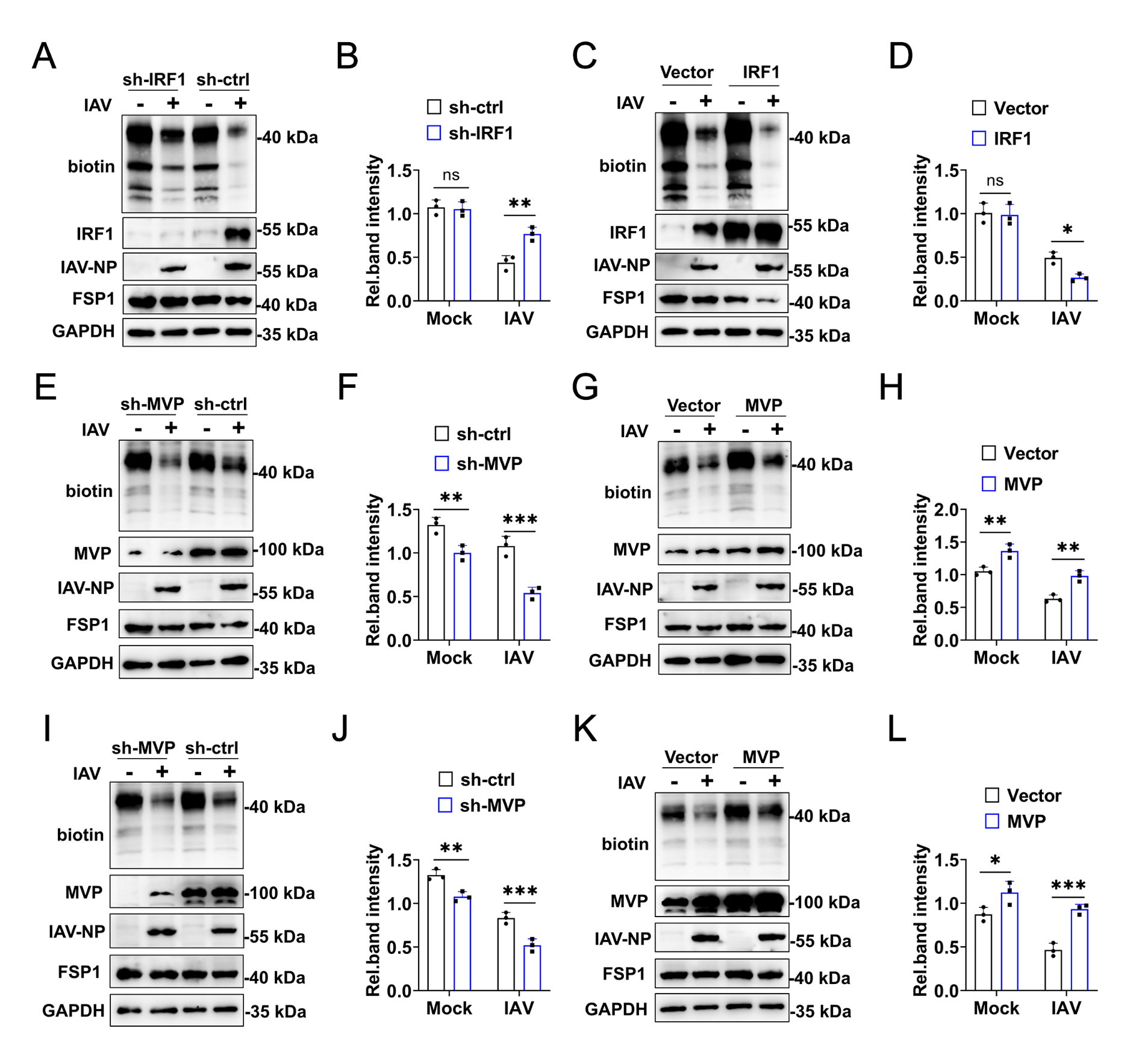


**Figure S10:** MVP and IRF1 directly regulate the myristoylation of FSP1.

(A, B) A549 cells were transfected with sh-ctrl or sh-IRF1 for 36 hrs. Then, sh-ctrl transfected cells were infected with IAV (MOI=0.1) and sh-IRF1 transfected cells were infected with IAV (MOI=0.04) for 12 hrs before click chemistry analyses (A). The relative intensity of myristoylation of FSP1 was measured using ImageJ and normalized to the respective unmodified FSP1 (B).

(C, D) A549 cells were transfected with vector-ctrl or pCMV-IRF1 for 36 hrs. Then, vector-ctrl transfected cells were infected with IAV (MOI=0.02) and pCMV-IRF1 transfected cells were infected with IAV (MOI=0.1) for 12 hrs before click chemistry analyses (C). The relative intensity of myristoylation of FSP1 was measured using ImageJ and normalized to the respective unmodified FSP1 (D).

(E-H) Vero cells were transfected with the indicated plasmids or shRNAs for 36 hrs and infected with or without IAV (MOI = 0.1) for 12 hrs, followed by click chemistry analyses. The quantification of the Western blots is shown in the adjacent graphs.

(I, J) A549 cells were transfected with sh-ctrl or sh-MVP for 36 hrs. Then, sh-ctrl transfected cells were infected with IAV (MOI=0.09) and sh-MVP transfected cells were infected with IAV (MOI=0.05) for 12 hrs before click chemistry analyses (I). The relative intensity of myristoylation of FSP1 was measured using ImageJ and normalized to the respective unmodified FSP1 (J).

(K, L) A549 cells were transfected with vector-ctrl or pCMV-MVP for 36 hrs. Then, vector-ctrl transfected cells were infected with IAV (MOI=0.05) and pCMV-MVP transfected cells were infected with IAV (MOI=0.09) for 12 hrs before click chemistry analyses (K). The relative intensity of myristoylation of FSP1 was measured using ImageJ and normalized to the respective unmodified FSP1 (L).

We acknowledge the use of GraphPad Prism 8.0 and Adobe Photoshop CC2019 for generating this figure. All experiments were performed in triplicate. The data are presented as mean ± SD. Statistical significance was assessed using a two-way ANOVA. *p < 0.05; **p < 0.01; ***p < 0.001. n.s. = not significant.

**Table S1.** The characteristics of IAV patients and healthy individuals (Cohort #1)

| IAV patients | | | Healthy individuals | | |
| --- | --- | --- | --- | --- | --- |
| Number | Age | Gender | Number | Age | Gender |
| 1 | 35 | Male | 1 | 26 | Female |
| 2 | 28 | Female | 2 | 42 | Male |
| 3 | 46 | Male | 3 | 21 | Male |
| 4 | 21 | Male | 4 | 64 | Female |
| 5 | 63 | Female | 5 | 36 | Female |
| 6 | 38 | Male | 6 | 34 | Male |
| 7 | 44 | Female | 7 | 55 | Female |
| 8 | 25 | Female | 8 | 49 | Male |
| 9 | 66 | Female | 9 | 42 | Male |
| 10 | 41 | Male | 10 | 18 | Female |
| 11 | 28 | Male | 11 | 23 | Male |
| 12 | 19 | Female | 12 | 33 | Female |
| 13 | 32 | Male | 13 | 31 | Female |
| 14 | 38 | Female | 14 | 54 | Male |
| 15 | 52 | Male | 15 | 46 | Male |
| 16 | 31 | Male | 16 | 61 | Male |
| 17 | 61 | Female | 17 | 52 | Female |
| 18 | 45 | Female | 18 | 22 | Female |
| 19 | 33 | Male | 19 | 31 | Male |
| 20 | 20 | Female | 20 | 30 | Female |
| 21 | 39 | Female |  |  |  |
| 22 | 31 | Male |  |  |  |
| 23 | 46 | Female |  |  |  |
| 24 | 37 | Male |  |  |  |

**Table S2.** The characteristics of IAV patients and healthy individuals (Cohort #2)

| IAV patients | | | Healthy individuals | | |
| --- | --- | --- | --- | --- | --- |
| Number | Age | Gender | Number | Age | Gender |
| 1 | 22 | Male | 1 | 36 | Male |
| 2 | 28 | Female | 2 | 27 | Female |
| 3 | 35 | Female | 3 | 44 | Male |
| 4 | 26 | Male | 4 | 62 | Female |
| 5 | 52 | Female | 5 | 21 | Male |
| 6 | 39 | Male | 6 | 37 | Male |
| 7 | 24 | Male | 7 | 42 | Female |
| 8 | 43 | Female | 8 | 31 | Male |
| 9 | 46 | Male | 9 | 51 | Male |
| 10 | 67 | Male | 10 | 28 | Female |
| 11 | 28 | Female | 11 | 36 | Male |
| 12 | 33 | Female | 12 | 35 | Female |
| 13 | 29 | Male | 13 | 43 | Male |
| 14 | 45 | Male | 14 | 22 | Female |

**Table S3.** Antibodies and Reagents

| Reagent | Source | Identifier |
| --- | --- | --- |
| **Antibodies** | | |
| anti-4HNE | Abcam | ab46545 |
| anti-HA | Sigma | H6533 |
| anti-Flag | Sigma | F3165 |
| anti-Myc | Thermo | AH00052 |
| anti-GFP | Abcam | ab6556 |
| anti-ubiquitin | CST | 3936 |
| anti-IRF1 | Proteintech | 11335-1-AP |
| anti-MVP | Proteintech | 16478-1-AP |
| anti-FSP1 | Proteintech | 20886-1-AP |
| anti-GPX4 | Proteintech | 67763-1-Ig |
| anti-SLC40A1 | Proteintech | 26601-1-AP |
| anti-SLC7A11 | ABclonal | A25302 |
| anti-TRAF6 | ABclonal | A23385 |
| anti-IAV NP | Santa Cruz | XY-1261M |
| anti-GAPDH | Proteintech | 60004-1-Ig |
| Mouse IgG | Proteintech | B900620 |
| **Chemicals** | | |
| 4-chlorobenzoic acid | Sigma | 135585 |
| iFSP1 | MedChemExpress | HY-136057 |
| Corn oil | Sigma | 8001-30-7 |
| BODIPY 581/591 C11 | ThermoFischer | D3861 |
| Ferrostatin-1 | MedChemExpress | HY-100579 |
| Z-VAD-FMK | MedChemExpress | HY-16658B |
| Necrostatin-1 | MedChemExpress | HY-15760 |
| azide myristic acid | MedChemExpress | HY-151855 |
| biotin-PEG4-alkyne | MedChemExpress | HY-140922 |
| TCEP | MedChemExpress | HY-107859 |
| TBTA | MedChemExpress | HY-116677 |
| DMEM | Gibco | 11965092 |
| F12K | Gibco | 21127022 |
| Resazurin sodium | MedChemExpress | HY-111391 |
| NADH | MedChemExpress | HY-F0001 |
| Ficoll | TBD | LTS1077 |
| TRIzol | Takara | T9108 |
| Propidium iodide | Yeasen | 40710ES03 |
| DAPI | Yeasen | 40728ES03 |
| Chloroquine | MedChemExpress | HY-17589A |
| MG-132 | MedChemExpress | HY-13259 |
| DiI | MedChemExpress | HY-D0083 |
| DDD85646 | MedChemExpress | HY-103056 |
| HRP-Streptavidin | Sigma | RABHRP3 |
| Lipofectamine 2000 | Thermo Fisher | 11668019 |
| **Kit** |  |  |
| Iron assay kit | Abcam | ab83366 |
| Glutathione Assay Kit | Sigma | CS0260 |
| SYBR-GREEN One-Step kits | Bio-Rad | 1725150 |
| BCA Assay Kit | Bio-Rad | 5000001 |
| Dual luciferase reporter assay system | Promega | E1910 |
| HRP-DAB kit | ZsBio | ZLI-9019 |
| Mycoplasma Detection Kit | Yeasen | 40612ES60 |
| CCK8 kit | Vazyme | A311 |

**Table S4.** shRNAs used in this study

| Oligonucleotides | Source | Identifier |
| --- | --- | --- |
| Sh-MVP-1 | Sigma-Aldrich | TRCN0000148563 |
| Sh-MVP-2 | Sigma-Aldrich | TRCN0000150124 |
| Sh-MVP-3 | Sigma-Aldrich | TRCN0000180269 |
| Sh-IRF1-1 | Sigma-Aldrich | TRCN0000014672 |
| Sh-IRF1-2 | Sigma-Aldrich | TRCN0000014668 |
| Sh-IRF1-3 | Sigma-Aldrich | TRCN0000014669 |
| Sh-FSP1-1 | Sigma-Aldrich | TRCN0000064423 |
| Sh-FSP1-2 | Sigma-Aldrich | TRCN0000064424 |
| Sh-FSP1-3 | Sigma-Aldrich | TRCN0000064425 |
| Sh-TRIM21-1 | Sigma-Aldrich | TRCN0000003983 |
| Sh-TRIM21-2 | Sigma-Aldrich | TRCN0000003986 |
| Sh-TRIM21-3 | Sigma-Aldrich | TRCN0000010839 |
| Sh-NMT2-1 | Sigma-Aldrich | TRCN0000035672 |
| Sh-NMT2-2 | Sigma-Aldrich | TRCN0000035673 |
| Sh-NMT2-3 | Sigma-Aldrich | TRCN0000291915 |

**Table S5.** Real-time PCR primers

| Gene name | 5’ primer (5’-3’) | 3’ primer (5’-3’) |
| --- | --- | --- |
| MVP(h) | GGGTGAGAGTTCCCCATCTG | GGCTCACAAGAAGATGACTGGT |
| IRF1(h) | CATGCCCTCCACCTCTGAAG | CCATCCACGTTTGTTGGCTG |
| GPX4(h) | GAGGCAAGACCGAAGTAAACTAC | CCGAACTGGTTACACGGGAA |
| ACSL4(h) | TCTGCTTCTGCTGCCCAATT | CGCCTTCTTGCCAGTCTTTT |
| SLC7A11(h) | ATGCAGTGGCAGTGACCTTT | GGCAACAAAGATCGGAACTG |
| FSP1(h) | GTGAGCAATCTGGAGGAGC | GGACAGGAGGAACGTCAGT |
| SLC40A1(h) | CTACTTGGGGAGATCGGATGT | CTGGGCCACTTTAAGTCTAGC |
| CHAC1(h) | GGTGACGCTCCTTGAAGATCAT | TCAGTGGTTGGTCAGGAGCAT |
| GAPDH(h) | GGAAGGTGAAGGTCGGAGTCAACGG | CTCGCTCCTGGAAGATGGTGATGGG |
| PTGS2(m) | CTGCGCCTTTTCAAGGATGG | GGGGATACACCTCTCCACCA |
| GAPDH(m) | AGGAGAGTGTTTCCTCGTCC | TGCCGTGAGTGGAGTCATAC |
| IAV-NP | ATCAGACCGAACGAGAATCCAGC | GGAGGCCCTCTGTTGATTAGTGT |
